# Single‐Cell Landscape of Bronchoalveolar Lavage Fluid Identifies Specific Neutrophils during Septic Immunosuppression

**DOI:** 10.1002/advs.202406218

**Published:** 2025-01-30

**Authors:** Rong Shen, Yi Jiang, Guanglong Liu, Shenjia Gao, Hao Sun, Xinyi Wu, Jiahui Gu, Han Wu, Ke Mo, Xing Niu, Ronen Ben‐Ami, Wanjing Shang, Jie Zhang, Jun Wang, Changhong Miao, Zhizhang Wang, Wankun Chen

**Affiliations:** ^1^ Department of Pathology Nanfang Hospital School of Basic Medical Sciences Southern Medical University Guangzhou Guangdong 510515 China; ^2^ Guangdong Province Key Laboratory of Molecular Tumor Pathology Guangzhou Guangdong 510515 China; ^3^ Department of Anesthesiology Zhongshan Hospital Fudan University Shanghai 200032 China; ^4^ Shanghai Key laboratory of Perioperative Stress and Protection Shanghai 200032 China; ^5^ Experimental Center of BIOQGene YuanDong International Academy Of Life Sciences Hong Kong 999077 China; ^6^ Infectious Diseases Unit Tel Aviv Sourasky Medical Center Faculty of Medicine Tel Aviv University Tel Aviv 6997801 Israel; ^7^ Lymphocyte Biology Section Laboratory of Immune System Biology National Institute of Allergy and Infectious Diseases, NIH Bethesda MD 20814 USA; ^8^ Department of Integrative Medicine and Neurobiology School of Basic Medical Science Institutes of Integrative Medicine State Key Laboratory of Medical Neurobiology and MOE Frontiers Center for Brain Science Institutes of Brain Science Shanghai Medical College Fudan University Shanghai 200032 China; ^9^ Department of Anesthesiology Shanghai Geriatric Medical Center Shanghai 201104 China; ^10^ Department of Anesthesiology QingPu Branch of Zhongshan Hospital Affiliated to Fudan University Shanghai 201700 China

**Keywords:** bronchoalveolar lavage fluid, CXCR2, neutrophils, sepsis, single‐cell RNA sequencing

## Abstract

Sepsis‐induced immunosuppression is related to increased susceptibility to secondary infections and death. Lung is the most vulnerable target organ in sepsis, but the understanding of the pulmonary immunosuppression state is still limited. Here, single‐cell RNA sequencing of bronchoalveolar lavage fluid (BALF) is performed to map the landscape of immune cells, revealing a neutrophil‐driven immunosuppressive program in the lungs of patients with immunosuppressive sepsis. Although immunosuppressive genes are upregulated in different immune cells, only neutrophils dramatically increase in the BALF of patients in immunosuppressive phase of sepsis. Five neutrophil subpopulations in BALF are identified, among which *CXCR2* ^+^ and *CD274 (*PD‐L1 coding gene)^+^ *IL1RN* ^+^ neutrophil subpopulations increased significantly during septic immunosuppression. Interestingly, a developmental trajectory from *CXCR2* ^+^ to *CD274* ^+^ *IL1RN* ^+^ neutrophil subpopulation is disclosed. Moreover, the therapeutic effect of *CXCR2* blockade is observed on the survival of septic mice, along with a decreased number of PD‐L1*^+^* neutrophils. Taken together, the *CXCR2* ^+^ neutrophil subpopulation is discovered as a contributor to immunosuppression in sepsis and identified it as a potential therapeutic target in sepsis treatment.

## Graphical abstract

Lung is the most susceptible target organ in sepsis, here, the pulmonary immune landscape of immunosuppressive sepsis patients is showed through scRNA‐seq. The *CXCR2^+^* neutrophil subpopulation can be recruited by *CXCL8*, and further differentiate into the immunosuppressive *CD274^+^IL1RN^+^* subpopulation in lung. *CXCR2^+^* neutrophils is highlighted as a contributor to septic immunosuppression and a potential therapeutic target in sepsis.

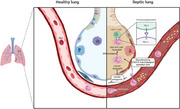

## 1 Introduction

Sepsis is considered as dysregulated responses upon infection, leading to life‐threatening organ dysfunction, followed by acute and persistent immunosuppression, which is implicated as a predisposing factor for the patients’ increased susceptibility to secondary infections and death.^[^ ^1^, ^2^ ^]^ With advances in intensive care medicine and goal‐directed interventions, most patients survive the initial hyper‐inflammatory phase of sepsis but progress to the latter long‐term immunosuppression stage.^[^ ^3^, ^4^, ^5^ ^]^ In particular, the onset of immunosuppression is related to a high risk of pneumonia and respiratory dysfunction.^[^ ^6^ ^]^ As the most frequently affected organ in sepsis, pulmonary dysfunction is closely associated with patients’ prognosis.^[^ ^2^, ^7^, ^8^, ^9^, ^10^ ^]^ However, understanding of the component and role of immune cells in the lung environment during the immunosuppression stage of sepsis is limited.

Neutrophils are important immune effector cells constituting the first‐line defense against pathogens. During bacterial and viral infections, neutrophils are rapidly recruited to the sites of inflammation, recognize and phagocytose microorganisms, and then kill pathogens through a combination of cytotoxic mechanisms.^[^ ^11^, ^12^ ^]^ In addition to immune defense, neutrophils participate in multiple pathophysiological processes. For instance, aberrant neutrophil activation contributes to cancer, autoimmune diseases and other inflammatory conditions.^[^ ^13^ ^]^ Previous studies have found that neutrophils are expanded and play an important role in the progression of sepsis.^[^ ^14^, ^15^, ^16^, ^17^ ^]^ Neutrophil dysfunction, as well as impaired migration to distant vital organs and immunosuppressive state, is associated with sepsis morbidity and mortality.^[^ ^14^, ^15^, ^16^, ^17^ ^]^

Here, we performed single‐cell RNA sequencing (scRNA‐seq) on bronchoalveolar lavage fluid (BALF) from patients in immunosuppressive phase of sepsis and healthy donors. Clustering analysis identified five neutrophil subpopulations in BALF, among which *CXCR2* ^+^ and *CD274* ^+^ *IL1RN* ^+^ subpopulations increased significantly in septic patients compared to healthy donors. Further analysis revealed the developmental trajectory from the *CXCR2* ^+^ subpopulation (at lower maturation status and with higher migration capability) to the *CD274* ^+^ *IL1RN* ^+^ subpopulation (with immunosuppressive function). Meanwhile, an enhancement of *CXCL8*‐*CXCR2* interaction between *CXCR2* ^+^ subpopulation and *CXCL8*‐expressing macrophages / epithelial cells was observed, suggesting the notion that *CXCR2* ^+^ neutrophils can be recruited by *CXCL8* and further transit into *CD274* ^+^ *IL1RN* ^+^ neutrophils. Finally, the therapeutic effect of *CXCR2* blockade on the survival of septic mice was determined. These findings suggest that the *CXCR2* ^+^ neutrophil subpopulation may serve as a contributor to immunosuppression in sepsis.

## 2 Results

### 2.1 Neutrophils Dramatically Increase in Sepsis Patients’ BALF

**Figure 1:**
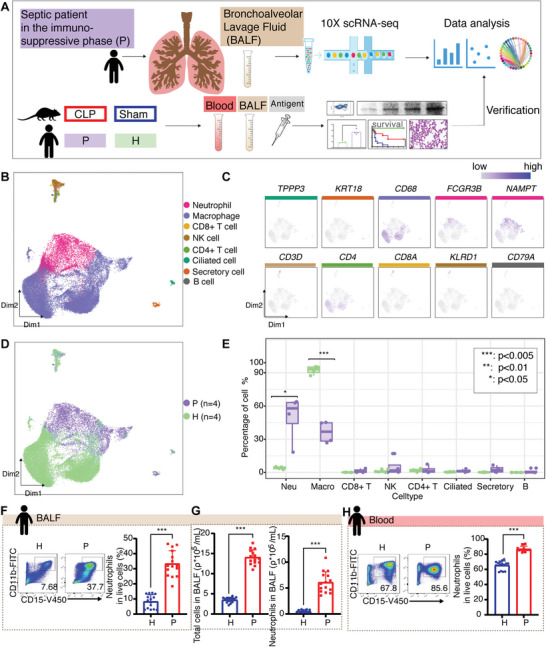
Cell composition alterations in peripheral blood and BALF of sepsis patients. A) Experimental design of the study. B) UMAP presentation of major cell types. C) Novel marker expression of major cell types. D) UMAP presentation of the sample group by disease status. E) Proportion of cell type composition change in patient and control samples. Asterisks denote significant changes (* denotes *p* < 0.05, ** denotes *p* < 0.01, *** denotes *p* < 0.005, by Student's t test). F) Flow cytometry for analyzing the proportion of neutrophils in BALF of patients and controls (for each group, *n* = 15). G) Total cell counts and neutrophil counts in BALF of patients and controls (for each group, *n* = 15). H) The proportion of neutrophils in peripheral blood of patients and controls (for each group, *n* = 15). For Figure 1F–H, *** denotes *p* < 0.001, by Student's t test.

To characterize the cellular composition in immunosuppressive stage of sepsis, we performed scRNA‐seq on BALF from four immunosuppressive sepsis patients (P1, P2, P3, P4). To compare the absolute frequency of these cell types between sepsis patients and healthy controls, we referred to the cell composition of three external healthy control BALF samples (H2, H3, H4) from a public database,^[^ ^18^ ^]^ and to validate the reliability of controls, we additionally sequenced a healthy control (H1) to make a total of four healthy controls in our analysis (H1, H2, H3, H4). Figure S1A–C (Supporting Information) displays the identification indicators of sepsis‐related immunosuppression in patients and healthy controls. It is worth noting that drugs such as ulinastatin^[^ ^19^ ^]^ and glucocorticoid may have immunomodulatory effects in sepsis patients. To ensure that the BALF samples obtained reflect the immune responses in the lungs of sepsis patients, we excluded those using immunomodulatory drugs and glucocorticoid from the study. The study design is shown in **Figure** **1**A, and sample information is listed in Tables S1 and S2 (Supporting Information). After preprocessing and quality control, we obtained an average of 1518 unique transcripts per cell from a total of 43854 cells, with 7269 cells from sepsis patients and 36585 cells from healthy controls. Hierarchical clustering generated 26 distinct uniform manifold approximation and projection (UMAP) clusters (Figure S1D–F, Supporting Information). Based on the expression of known markers, they were annotated as six major cell types: macrophages (*CD68*), neutrophils (*FCGR3B*), T cells (*CD3D*), NK cells (*KLRD1*), epithelial cells (*TPPP3*, *KRT18*), and B cells (*MS4A1, CD79A*) (Figure 1B,C; Figure S1G,H, Supporting Information). In immunosuppressive sepsis patients, compared to healthy controls, the proportion of neutrophils in sepsis patients was significantly increased, while the proportion of macrophages was significantly decreased in patients (Figure 1D,E). No significant difference was observed in other cell types (Figure 1D,E).

In line with the above single‐cell data, the proportion and absolute number of neutrophils in BALF from sepsis patients in the immunosuppressive phase were significantly higher than that from controls (Figure 1F,G), along with increased total cells in septic BALF (Figure 1G). We also observed a moderate elevation in the proportion of blood neutrophils in sepsis patients compared to controls (Figure 1H).

**Figure 2:**
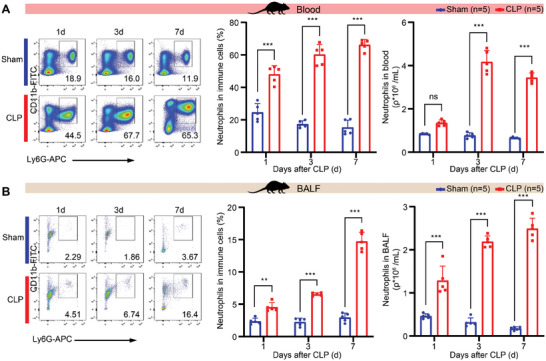
Change of neutrophils in peripheral blood and BALF in sepsis mouse model. A) The proportion and number of neutrophils in peripheral blood of CLP mice and Sham mice 1, 3, or 7 days post‐operation (for each group from each time point, *n* = 5). B) The proportion and number of neutrophils in BALF of CLP mice and Sham mice 1, 3, or 7 days post‐operation (for each group from each time point, *n* = 5). ns denotes not significant, ** denotes *p* < 0.01, *** denotes *p* < 0.001. Two‐way ANOVA was used for Figure 2A,B.

In immunosuppressed sepsis mice (CLP), relative body weight and survival rate decreased, while BALF colony‐forming units and total cells increased (Figure S1I‐L, Supporting Information) following a similar trend.^[^ ^20^ ^]^ CD4^+^ and CD8^+^ T cells in peripheral blood were reduced, while regulatory T cells increased, and lymphocyte and monocyte HLA‐DR levels were lowered (Figure S1M‐Q, Supporting Information), consistent with the immunosuppressive state observed in sepsis patients (Figure S1A‐C, Supporting Information).   The proportions of blood and BALF neutrophils were increased after CLP and reached the highest value at day 7 post CLP (**Figure** **2**A,B), indicating that neutrophils participate in the immunosuppressive phase of sepsis. Our findings are consistent with previous studies, which have shown that neutrophils are significantly increased in distant vital organs such as skeletal muscle and blood during sepsis.^[^ ^14^, ^15^, ^16^, ^17^, ^21^ ^]^

### 2.2 Immunosuppressive Genes are Upregulated in Neutrophils of Immunosuppressive Sepsis Patients

The immunosuppressive phase of sepsis is characterized by altered expression levels of immunosuppressive‐related genes such as *CD274* (PD‐L1) and *HLA‐DR*.^[^ ^2^, ^22^, ^23^ ^]^ To identify genes regulating immunosuppression in neutrophils during septic immunosuppression, we profiled the transcriptomic alterations of the above major six cell types in immunosuppressive sepsis patients and healthy controls (Figure S2A–C, Supporting Information). Among different cell groups, the number of differentially expressed genes (DEGs) corresponding to sepsis varied greatly. Overall, neutrophils, macrophages and secretory epithelial cells had more differences in gene expression than other cell types (Figure S2A, Supporting Information).

**Figure 3:**
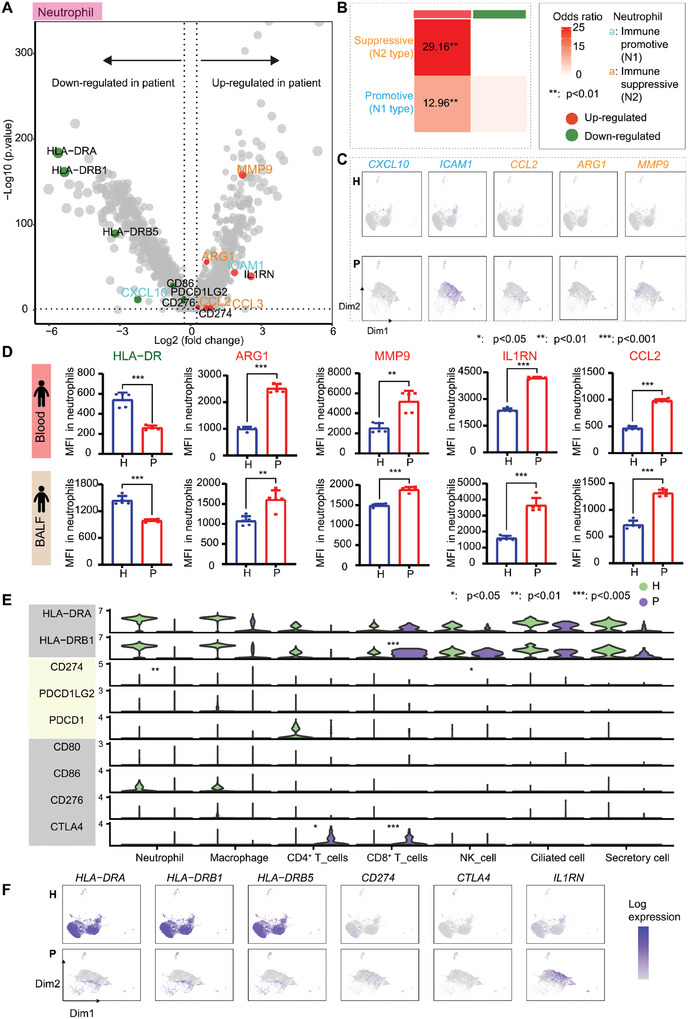
Upregulation of immunosuppressive genes in neutrophils of immunosuppressive sepsis patients. A) Differential expression in septic patient and control neutrophils. Upregulated/downregulated genes are shown as red/green points (dashed lines indicate p value = 0.05 and fold‐change = 1.2, wilcox test). B) Enrichment of upregulated/downregulated genes in immunopromotive (N1) or immunosuppressive (N2) types of neutrophils. Color and number denote the enrichment score (Fisher's exact test, odds ratio>1, ** *p* < 0.01). Immunopromotive/immunosuppressive types of neutrophils are shown in blue/orange. C) UMAP presentation of immunopromotive (in orange) or immunosuppressive (in blue) type neutrophil‐related genes in healthy controls (H) and patients (P). D) HLA‐DR/ARG1/IL1RN/MMP9/CCL2 MFI in neutrophils in peripheral blood and BALF in patients and controls (for each group, *n* = 5; * denotes *p* < 0.05, ** denotes *p* < 0.01, *** denotes *p* < 0.001, by Student's t test). E) Expression of immunosuppression‐related genes (MHC‐II pathway, PD‐L1 pathway, CTLA pathway) in different cell types in patients and controls (* denotes *p* < 0.05, ** denotes *p* < 0.01, *** denotes *p* < 0.005, by Student's t test). F) UMAP presentation of significantly changed immunosuppression‐related genes.

Although the RNA content of neutrophils was relatively low due to cell type properties, we still detected a substantial number of DEGs in sepsis patients, including decreased expression of genes related to the MHC‐II pathway (*HLA‐DR*) and increased expression of genes involved in immunosuppression, such as *ARG1*, *CD274* and *IL1RN* (**Figure** **3**A,E). Immune‐promotive N1 neutrophils are characterized by their pro‐inflammatory properties, while immunosuppressive N2 neutrophils are characterized by their immunosuppressive properties and express surface markers such as programmed death ligand 1 (PD‐L1), ARG1, and MMP9.^[^ ^24^, ^25^, ^26^ ^]^ Particularly, the expression of genes specifically regulating the immunosuppressive function of neutrophils, including *CD274*, *ARG1*, *MMP9*, and *CCL2*, were dramatically enhanced in neutrophils from sepsis patients compared to those from healthy controls (Figure 3A–C). In contrast, the expression of genes governing immune‐promoting function of neutrophils was decreased or had no significant change, except for *ICAM1*, which displayed an increased expression in sepsis patients but a smaller change compared with suppressive signature genes (Figure 3A–C). To verify this result, we collected and measured the MFI of neutrophil HLA‐DR/ARG1/IL1RN/MMP9/CCL2 in peripheral blood and BALF of sepsis patients, and found that consistent with the result, the level of HLA‐DR was significantly decreased, while the levels of ARG1/IL1RN/MMP9/CCL2 were significantly increased (Figure 3D).

The altered expression of immunoregulatory genes was also observed in other cell types. The upregulation of *CTLA4* in T cells and the upregulation of *CD274* in neutrophils indicated that the regulatory network of the immune system underwent complex changes, resulting in a weakened immune response. Besides, *HLA‐DR* was decreased in almost all cell types of sepsis patients (Figure 3E,F), potentially weakening the adaptive immune response. In addition, GO analysis revealed that the upregulated genes in neutrophils of sepsis patients included a mix of immune‐promotive and immunosuppressive genes that enhanced innate immunity and inflammatory response and inhibited adaptive immune responses and antigen presentation, respectively (Figure S2B, Supporting Information).

Collectively, these data reveal that neutrophils in immunosuppressive sepsis patients undergo dynamic genomic expression alterations, specified by higher expression of immunosuppressive genes related to adaptive immune response, implying that neutrophils may play an important role in immunosuppression of sepsis.

### 2.3 Heterogeneity Exists in Neutrophils of Immunosuppressive Sepsis Patients

**Figure 4:**
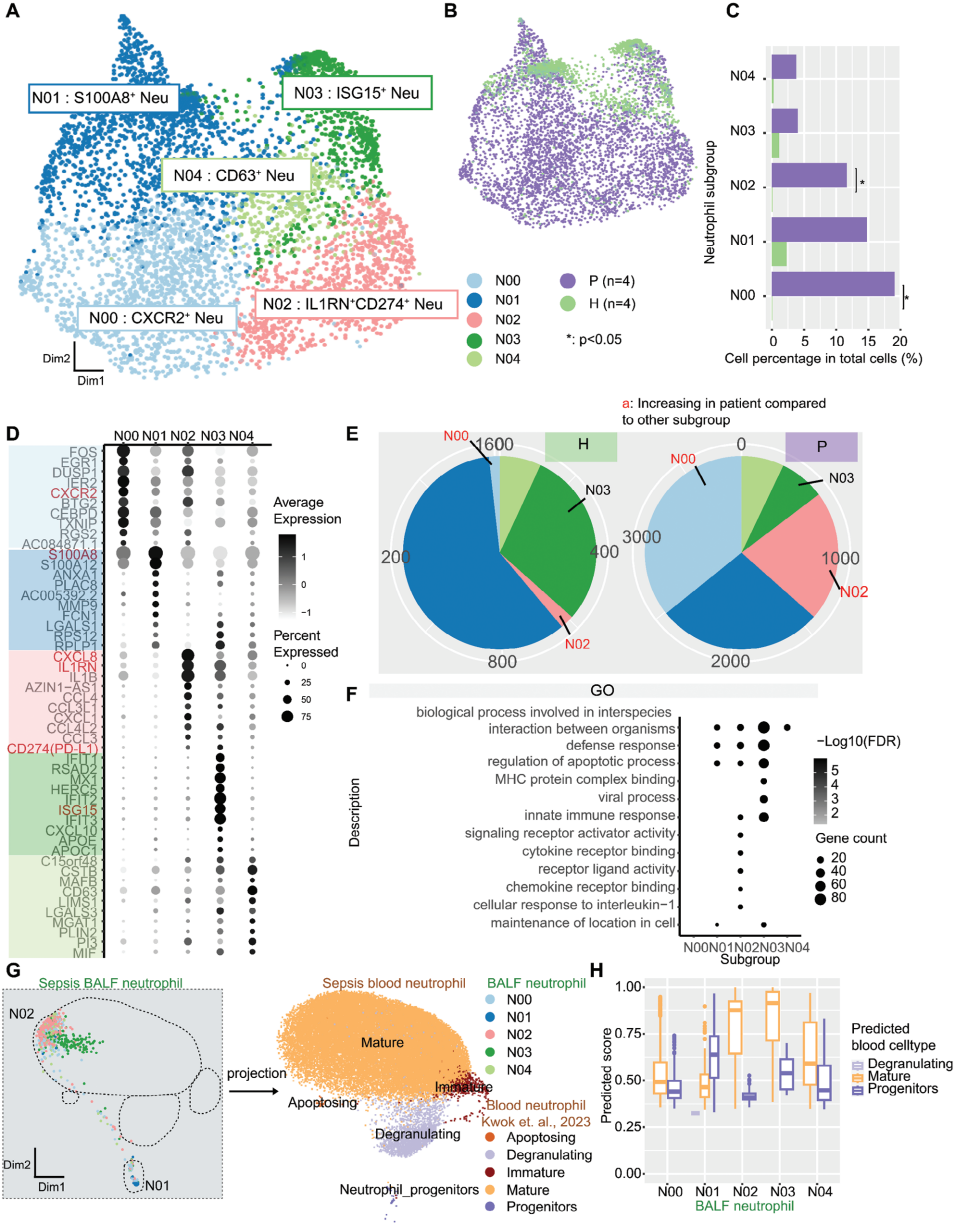
Heterogeneity of neutrophils in sepsis patient BALF. A,B) UMAP presentation of neutrophil clusters N00‐N04 and specific markers among all samples (A) and patient (P) and healthy control (H) BALF samples (B). C) Cell percentage of the neutrophil subgroups in control (green bar) and patient BALF samples (purple bar) (* denotes *p* < 0.05, Student's t test). D) Signature genes of neutrophil clusters N00‐N04. E) Relative composition of neutrophil subgroups in control (green bar) and patient (purple bar) BALF samples. Red characters denote subgroups with increased relative neutrophil composition in patients. F) Top enriched Gene Ontology (GO) terms in neutrophil clusters. G,H) Projection of sepsis BALF neutrophil subpopulations to sepsis blood neutrophil subpopulations from a public dataset ^[^ ^17^ ^]^. (G) UMAP projection of sepsis BALF neutrophil subpopulations onto sepsis blood neutrophil subpopulations. The dotted line on the UMAP of BALF neutrophils indicates the corresponding position to the UMAP of blood neutrophils. Colors denote the original subpopulation annotations. H) Prediction score of each sepsis BALF neutrophil subpopulation to sepsis blood neutrophil subpopulations.

To elucidate neutrophil heterogeneity and identify potential neutrophil subpopulations, we re‐clustered 4103 neutrophils from sepsis patients and controls based on gene expression differences. We identified five subpopulations in BALF neutrophils from immunosuppressive sepsis patients: N00 (*CXCR2* ^+^), N01 (*S100A8/12* ^+^), N02 (*CD274^+^IL1RN^+^*), N03 (*ISG15* ^+^), and N04 (*CD63* ^+^) (**Figure** **4**A,D). According to the co‐expression of *CD274* and *IL1RN*, N02 was defined as an immunosuppression‐related subpopulation. We next analyzed the maturation status of each neutrophil subpopulation by comparing with a neutrophil maturation dataset.^[^ ^27^ ^]^ N01 overlapped with the genes specific for immature neutrophils, while N00 and N02 had obvious overlaps with genes expressed in mature neutrophils, suggesting that the maturity of N01 may be lower than that of N00 and N02 (Figure S3A, Supporting Information).

Except for N03, the cell percentages of N00, N01, N02, and N04 were significantly increased in BALF of sepsis patients compared to controls (Figure 4B,C). Besides, the relative composition of *CXCR2* ^+^ N00 and *CD274* ^+^ *IL1RN* ^+^ N02 was also increased in immunosuppressive sepsis BALF (Figure 4E), indicating that the N00 and N02 neutrophil subpopulations might be more crucial to sepsis‐induced immunosuppression than other subpopulations. GO enrichment analysis revealed that the gene expression of N02 was enriched in signaling pathways such as signal receptor regulation activity and cytokine‐receptor binding (Figure 4F).

The absolute proportion of N03 subpopulation in patients changed weakly. Although the number of neutrophils in patients was increased compared to other cell types, the proportion of *ISG15* ^+^ N03 was decreased relative to the total neutrophil number. GO enrichment analysis showed that specific genes of N03 were involved in the viral infection pathway, indicating that N03 may be related to viral infection (Figure 4F). Since sepsis is mainly caused by bacterial infection, these results indicate that *ISG15* ^+^ N03 may be a viral‐related neutrophil subpopulation in BALF but did not contribute to the sepsis immunosuppressive signature.

In immunosuppressive stage of sepsis, neutrophils were significantly increased in blood, accompanied by differences among neutrophil subpopulations.^[^ ^17^ ^]^ We compared our neutrophil subpopulations (N00‐N04) with the reported clusters in peripheral blood of sepsis patients.^[^ ^28^ ^]^ We found that N01 was similar to their septic‐related Neu1 subtype, and N03 was similar to their Neu3 (Figure S3B, Supporting Information). Notably, N02 characterized by *IL1RN* and *CD274* was unique to BALF and absent in peripheral blood, suggesting a distinct population in the lung environment of sepsis patients. To clarify the connection of neutrophils in the BALF with neutrophils in plasma, we projected the BALF neutrophil data collected from immunosuppressive sepsis patients onto the plasma neutrophil data of community acquired pneumonia (CAP) sepsis patients.^[^ ^17^ ^]^ It was found that the neutrophils in BALF were mainly projected to mature neutrophils, neutrophil progenitors and degranulating neutrophils. First, BALF N01 neutrophils were mainly projected to neutrophil progenitors in blood, indicating that N01 neutrophils were most similar to neutrophil progenitors existing in blood (Figure 4G,H). N01 neutrophils existed both in healthy and sepsis BALF, with significant increase in cell number in sepsis BALF (Figure 4B,C,E), inferring an increase in neutrophil progenitor migration from blood to lung. Second, N00/N04 contained both mature neutrophil‐like cells and progenitor‐like cells (Figure 4G,H; Figure S3C, Supporting Information), in which the N00 content in sepsis was significantly increased with a higher relative content, indicating that this group of cells may be an intermediate state from progenitor to mature neutrophils and play an important role in the sepsis BALF environment. Finally, N02 and N03 were mainly projected into mature neutrophils (Figure 4G,H; Figure S3C, Supporting Information), which did not change significantly in blood and were actually different from mature neutrophils in the peseudotime analysis (Figure S3C, Supporting Information). The content of combined N02 in BALF was significantly increased (Figure 4B,C), suggesting that N02 is a distinct immunosuppressive neutrophil subpopulation differentiated in lung environment.

Differential expression analysis of neutrophils in immunosuppressive sepsis patients and healthy controls showed that DEGs of each subpopulation showed some similarities (Figure S3D–F, Supporting Information). For instance, *S100A8/12* were significantly upregulated in most of the subpopulations in patients, while *HLA‐DR* and other MHC‐II pathway genes were significantly downregulated in most of the subpopulations (Figure S3D, Supporting Information). GO and KEGG pathway enrichment analysis of DEGs revealed that both in N00 and N02, upregulated DEGs in patients were related to cell apoptosis, and downregulated DEGs were enriched in GO terms such as MHC‐II and antigen processing and presentation (Figure S3E, Supporting Information), suggesting that N00/N02 may share these molecular functions. However, *IL1RN*, the natural antagonist of *IL1B*, was only significantly upregulated in N02 (Figure S3D, Supporting Information), indicating that *IL1B* function may be suppressed in N02 subpopulation.

### 2.4 *CXCR2* ^+^ and *CD274* ^+^ *IL1RN* ^+^ Neutrophil Subpopulations Increase in Sepsis

**Figure 5:**
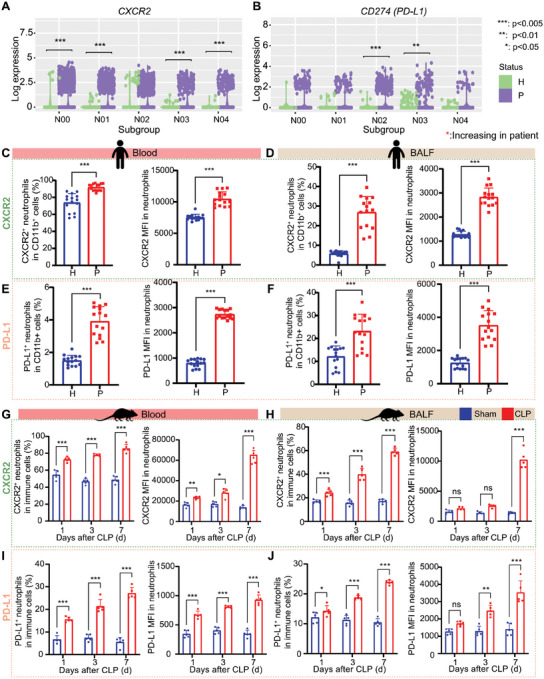
Increased *CXCR2^+^* neutrophils and *CD274* ^+^ *IL1RN* ^+^ neutrophils in sepsis. A) *CXCR2* expression in neutrophils N00‐N04. B) *CD274* (PD‐L1 coding gene) expression in neutrophils N00‐N04. C) The proportion of CXCR2^+^ neutrophils and CXCR2 MFI in peripheral blood of sepsis patients and controls (for each group, *n* = 15). D) The proportion of CXCR2^+^ neutrophils and CXCR2 MFI in BALF of patients and controls (for each group, *n* = 15). E) The proportion of PD‐L1^+^ neutrophils and PD‐L1 MFI in peripheral blood of patients and controls (for each group, *n* = 15). F) The proportion of PD‐L1^+^ neutrophils and PD‐L1 MFI in BALF of patients and controls (for each group, *n* = 15). G) The proportion of CXCR2^+^ neutrophils and CXCR2 MFI in peripheral blood of CLP mice and Sham mice on day 1/3/7 (*n* = 5 per group per time point). H) The proportion of CXCR2^+^ neutrophils and CXCR2 MFI in BALF of CLP mice and Sham mice on days 1/3/7 (*n* = 5 per group per time point). I) The proportion of PD‐L1^+^ neutrophils and PD‐L1 MFI in peripheral blood of CLP mice and Sham mice on day 1/3/7 (*n* = 5 per group per time point). J) The proportion of PD‐L1^+^ neutrophils and PD‐L1 MFI in BALF of CLP mice and Sham mice on day 1/3/7 (*n* = 5 per group per time point). For Figure 5A,B, * denotes *p* < 0.05, ** denotes *p* < 0.01, *** denotes *p* < 0.005, by wilcox test. For Figure 5C–J, ns denotes not significant, * denotes *p* < 0.05, ** denotes *p* < 0.01, *** denotes *p* < 0.001. Student's t test was used for for Figure 5C–F. Two‐way ANOVA was used for Figure 5G–J.

To reveal the possible functional diversity of these subpopulations, we first compared their gene expression profiles (**Figure** **5**A,B; Figure S3D, Supporting Information). Interestingly, although *CXCR2* was identified as the specific marker of N00, it was also upregulated in almost all neutrophil subpopulations except N02 in sepsis patients compared to controls (Figure 5A). *CD274*, the main receptor in the PD‐1 signaling pathway that enhances immune escape in tumors,^[^ ^29^ ^]^ was also upregulated slightly in the N02 subpopulation (avg_log2FC = 1.22, *p* < 0.001; Figure 5B). Furthermore, *IL1RN* was upregulated in the patients’ N02 subpopulation with a high fold‐change (avg_log2FC = 4.079; Figures S3D and S4E, Supporting Information), while *IL1B* was upregulated with a relatively lower fold‐change (avg_log2FC = 2.726288; Figures S3D and S4E, Supporting Information).

Consistent with the observation of the increase in *CXCR2* ^+^ and *CD274* ^+^ *IL1RN* ^+^ neutrophil subpopulations in sepsis, the amplification of *CXCR2^+^* and *PD‐L1* ^+^ subpopulations was observed in both blood and BALF neutrophils in immunosuppressive sepsis patients (Figure 5C–F; Figure S4A–D, Supporting Information). In additionally collected clinical samples, we observed the enhanced MFI of CXCR2 and PD‐L1 in both blood and BALF neutrophils (Figure 5C–F; Figure S4A–D, Supporting Information). Moreover, the amplification of CXCR2^+^ subpopulation and the enhanced CXCR2 MFI in neutrophils from peripheral blood and BALF in CLP‐induced sepsis mice were observed, especially in the immunosuppressive period (day 7) (Figure 5G,H). The expression of PD‐L1 also showed a pattern similar to that observed in humans (Figure 5I,J). The consistent observations in CLP mice and sepsis patients during the immunosuppressive phase indicated that not only the proportions of CXCR2^+^ and PD‐L1^+^ neutrophil subpopulations increased in sepsis, but also the expression of *CXCR2* and PD‐L1, suggesting that these two subpopulations may play an essential role in the mechanism of sepsis‐induced immunosuppression.

These data further provide evidence for the notion that *CXCR2* ^+^ N00 and *CD274* ^+^ *IL1RN* ^+^ N02 neutrophil subpopulations play major roles in immunosuppressive phase of sepsis. Through analysis of cell communication of IL‐1 pathway, we found that the IL‐1 signaling pathway was enhanced in patient samples compared with control samples (Figure S4E, Supporting Information), as well as the interaction intensity of *IL1B*‐*IL1R2* (Figure S4E–H, Supporting Information). Since *IL1RN* is the natural antagonist of *IL1B* and can competitively bind to the IL‐1 receptor to inhibit the IL‐1 pathway, high expression of *IL1RN* may play a critical role in suppressing the immune‐promoting effects of the IL‐1 pathway, thereby contributing to the immunosuppressive state observed in sepsis, suggesting the importance of the *CD274* ^+^ *IL1RN* ^+^ neutrophil subpopulation in regulating the IL‐1 pathway.

### 2.5 Differentiation of *CXCR2* ^+^ Neutrophils to Immunosuppressive *IL1RN* ^+^PD‐L1^+^ Neutrophils

**Figure 6:**
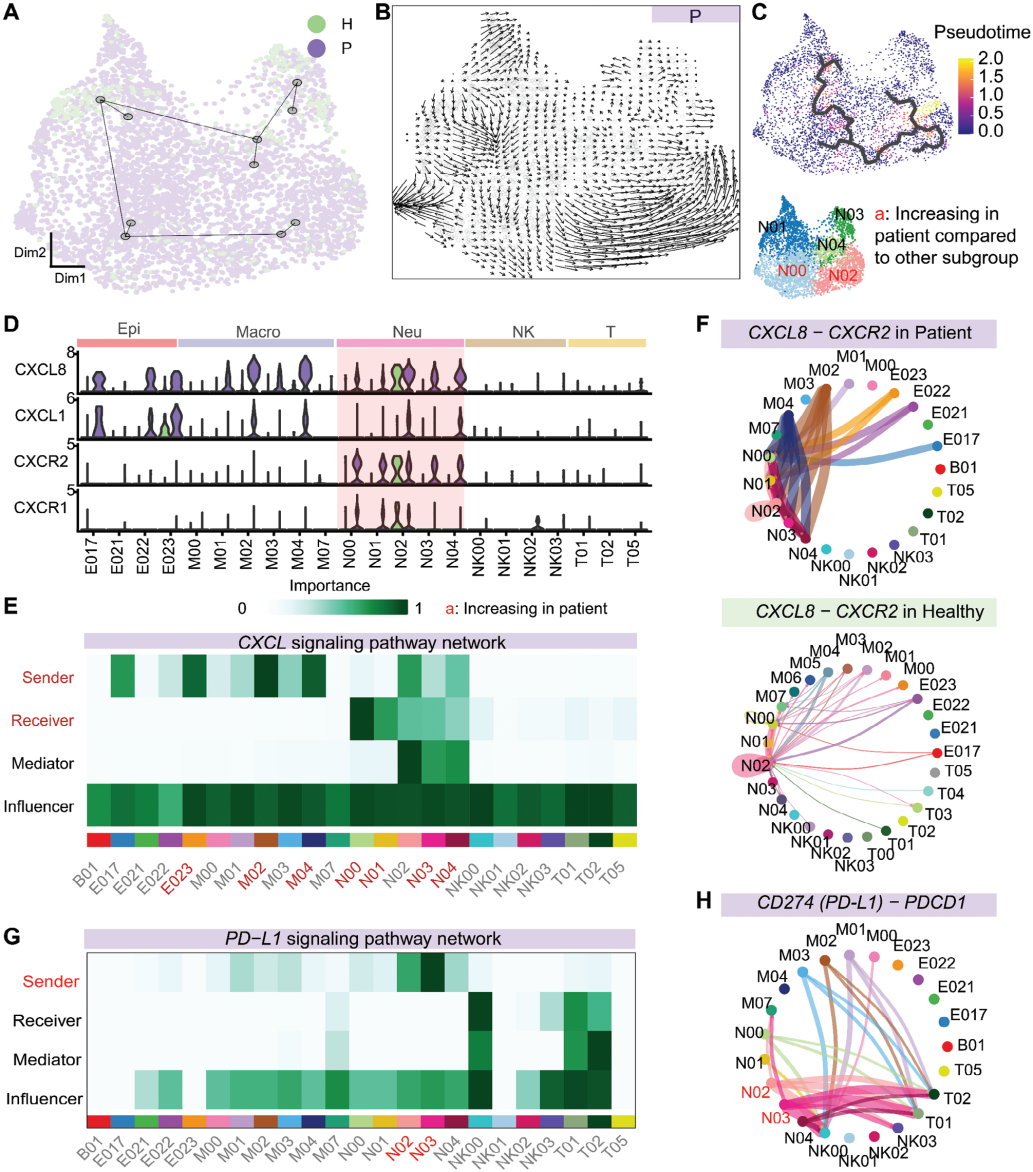
Differentiation of *CXCR2* ^+^ neutrophils to *IL1RN* ^+^ PD‐L1^+^ neutrophils. A) Trajectory inference analysis demonstrating the heterogeneity of neutrophil clusters (via Slingshot, using healthy cells as the root). B) RNA velocity analysis of neutrophil clusters. C) Pseudotime analysis demonstrating the heterogeneity of neutrophil clusters (via Monocle3, using healthy cells as the root). D) *CXCL8* (IL‐8 coding gene), *CXCL1*, and *CXCR1*/2 expression in different cell types, as shown in Figures S5B and S6C,F (Supporting Information). E) Changes in *CXCL* signaling pathway in patients (detailed in methods). Red characters denote increased signals in patients, as shown in Figures S5B,C and S6C,F (Supporting Information). F) Network diagram of *CXCR2*‐*CXCL8* signaling in patients and healthy controls, with interaction strength represented as the edge (detailed in methods). The purple bar denotes patient cell signaling, and the green bar denotes control cell signaling. G) Changes in PD‐1 pathway signaling in patients (detailed in methods). Red characters denote enhanced signaling in patients compared to healthy controls. H) Network diagram of *CD274*‐*PDCD1* (PD‐1 coding gene) signaling, with interaction strength represented as the edge (detailed in methods).

Next, we performed trajectory analysis on all neutrophil subpopulations to investigate the relationship of each subpopulation in immunosuppressive stage of sepsis. As a result, neutrophil subpopulations had a differentiation trajectory of *S100A8/12* ^+^ N01 → *CXCR2* ^+^ N00 → *CD274* ^+^ *IL1RN* ^+^ N02, suggesting that N02 might be the subpopulation that appeared in later differentiation stages (**Figure** **6**A, Slingshot based on the UMAP distance, described in methods). The peseudotime analysis also confirmed that N02 had a later peseudotime compared to other subpopulations (Figure 6C; Monocle3, described in methods). In addition, we used Velocyto to infer the differentiation direction of neutrophil subpopulations based on the RNA degradation rate, and also confirmed the trajectory of N00 → N02 (Figure 6B; Figure S5A, Supporting Information; described in methods). Taken together, these results suggest that neutrophil subpopulations display a trajectory transition from *CXCR2* ^+^ N00 to *CD274* ^+^ *IL1RN* ^+^ N02 in the lung environment during septic immunosuppression.

As shown above, N00 specifically expressed *CXCR2*, which is one of the receptors of the CXCL signaling pathway. Enhanced CXCL signaling pathway communication was observed in patients compared to controls (Figure S5B,C, Supporting Information), which might be related to increased ligand expression in macrophage *CXCL1^+^CXCL8^+^* M02/M04 subpopulations (Figure S6A–F, Supporting Information) and epithelial *KRT13* ^+^ E023 basel epithelial subpopulation (Figures S5B and S7A–F, Supporting Information) and increased receptor expression in neutrophils N00‐N04 (Figure 5D–F). Previous studies have shown that IL‐8 (encoded by *CXCL8*) and CXCL1 can bind to the chemokine receptors *CXCR1*/*CXCR2* to produce chemotaxis and neutrophil recruitment.^[^ ^30^, ^31^, ^32^, ^33^, ^34^ ^]^ Based on the analysis of the ligand‒receptor pairs of the CXCL pathway, we found that *CXCL8/1*‐*CXCR1/2* stood out from the other *CXCL* pathway ligand‒receptor pairs (Figure 6D–F; Figure S5B,C, Supporting Information), with *CXCL8‐CXCR2* displaying the most important change, followed by *CXCL1‐CXCR2* (Figure S5B,C, Supporting Information). Specifically, the expression of ligand *CXCL8/CXCL1* in macrophage subpopulations M02/M04 and epithelial subpopulation E023 and the receptor *CXCR2* in subpopulations N00 and N01 were enhanced in patients compared with controls (Figure 6D–F). Cell communication analysis of the PD‐1 pathway revealed that the connection in N02 of *CD274*‐*PDCD1* was enhanced in sepsis patients compared with healthy controls (Figure 6G,H). These data suggest that the N02 subpopulation may possess immunosuppressive function.

Altogether, in immunosuppressive sepsis patients, the *CXCR2* ^+^ neutrophil subpopulation might be related to *CXCR2‐*mediated neutrophil migration and thereby contribute to the dramatic increase in neutrophils, while N02 cells expressing *IL1RN* and *CD274* may act as a major immunosuppressive neutrophil subpopulation.

Due to the significant increase in *CXCR2* ^+^ neutrophils and specific expression of *CXCL8/CXCL1* in macrophages and epithelial cells in patients, we speculated that CXCL8/CXCL1 may interact with CXCR2 to regulate its chemotaxis and therefore contribute to the transition of *CXCR2* ^+^ N00 to *CD274* ^+^ *IL1RN* ^+^ N02 neutrophil subpopulation.

**Figure 7:**
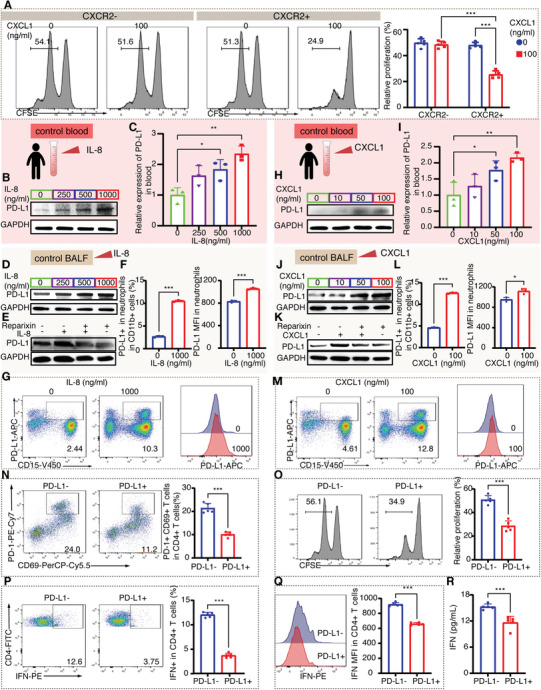
Immunosuppressive PD‐L1^+^ neutrophils regulated by IL‐8/CXCL1. A) Proliferation of CD4^+^ T cells after co‐culture with CXCR2^+^ or CXCR2^−^ neutrophils from healthy control peripheral blood with or without CXCL1 treatment (100 ng mL^−1^) for 72 h in vitro (for each group, *n* = 5). B–D) PD‐L1 expression in healthy control peripheral blood and BALF neutrophils (1×10^6^ mL^−1^) stimulated with recombinant human IL‐8 (250–1000 ng mL^−1^) (for each group, *n* = 3). E) PD‐L1 expression in BALF neutrophils stimulated with recombinant human IL‐8 (1000 ng mL^−1^) with or without Reparixin (1 mm) (for each group, *n* = 3). F,G) The proportion of PD‐L1^+^ neutrophils and PD‐L1 MFI in BALF neutrophils with the treatment of IL‐8 in vitro (for each group, *n* = 3). H–J) PD‐L1 expression in healthy control peripheral blood and BALF neutrophils (1×10^6^ mL) stimulated with recombinant human CXCL1 (10–100 ng mL^−1^) (for each group, *n* = 3). K) PD‐L1 expression in BALF neutrophils stimulated with recombinant human CXCL1 (100 ng mL^−1^) with or without Reparixin (1 mm) (for each group, *n* = 3). L,M) The proportion of PD‐L1^+^ neutrophils and PD‐L1 MFI in BALF neutrophils with the treatment of CXCL1 in vitro (for each group, *n* = 3). N) The proportion of PD‐1^+^ CD69^+^ T cells in CD4^+^ T cells co‐cultured with PD‐L1^+^ or PD‐L1^−^ neutrophils in vitro (for each group, *n* = 5). O) Proliferation of CD4^+^ T cells after co‐culture with PD‐L1^+^ or PD‐L1^−^ neutrophils for 72 h in vitro (for each group, *n* = 5). P,Q) IFN‐γ expression in CD4^+^ T cells co‐cultured with PD‐L1^+^ or PD‐L1^−^ neutrophils in vitro (for each group, *n* = 5). R) IFN‐γ levels in cell culture media co‐cultured with CD4^+^ T cells and PD‐L1^+^ or PD‐L1^−^ neutrophils in vitro (for each group, *n* = 5). * denotes *p* < 0.05, ** denotes *p* < 0.01, *** denotes *p* < 0.001. Two‐way ANOVA was used for Figure 7A. One‐way ANOVA was used for Figure 7C,I. Student's t test was used for for Figure 7F,L,N–R.

To prove this hypothesis, we sorted CXCR2^+^ and CXCR2^−^ neutrophils from peripheral blood and co‐cultured with CD4^+^ T cells with or without CXCL1 (100 ng mL^−1^) stimulation and found that under CXCL1 treatment, CXCR2^+^ neutrophils resulted in significant inhibition of CD4^+^ T cell proliferation compared to CXCR2^−^ neutrophils (**Figure** **7**A). However, without CXCL1 treatment, CXCR2^+^ neutrophils did not significantly affect CD4^+^ T cell proliferation. In conclusion, these results suggest that CXCL8/CXCL1 may regulate the immunosuppressive role of CXCR2^+^ neutrophil subpopulation. We stimulated peripheral blood and BALF neutrophils (1 × 10^6^ mL^−1^) isolated from healthy controls with recombinant human IL‐8 (250–1000 ng mL^−1^) or CXCL1 (10–100 ng mL^−1^). As a result, the expression of PD‐L1 was significantly upregulated in a dose‐dependent manner after both IL‐8 and CXCL1 treatment in blood and BALF neutrophils (Figure 7B–M). To test whether inhibition of IL‐8 and CXCL1 could downregulate the expression of PD‐L1 in BALF neutrophils, we selectively stimulated BALF neutrophils with recombinant human IL‐8 (1000 ng mL^−1^) or CXCL1 (100 ng mL^−1^) with or without Reparixin (1 mm). After the inhibition of IL‐8 and CXCL1 with Reparixin, the expression of PD‐L1 was downregulated (Figure 7E,K).

PD‐1 is an inhibitory molecule on CD4^+^ T cells. PD‐L1 can bind to PD‐1 and promote immunosuppression through inhibiting survival, proliferation and function of effector CD4^+^ T cells and promoting CD4^+^ T cells to differentiate into immunosuppressive phenotype.^[^ ^35^ ^]^ Therefore, we speculated that the increased expression of PD‐L1 in neutrophils may affect the differentiation and proliferation of CD4^+^ T cells, leading to immunosuppression in sepsis. Co‐culture with sorted PD‐L1^+^ neutrophils reduced the proportion of PD‐1^+^ CD69^+^ CD4^+^ T cells and CD4^+^ T cell proliferation (Figure 7N,O). In addition, we found that in CD4^+^ T cells co‐cultured with PD‐L1^+^ neutrophils, the expression of IFN was significantly reduced (Figure 7P–R). Together, these results indicate the enhanced immunosuppressive ability of PD‐L1^+^ neutrophils in vitro.

### 2.6 CXCR2 Antagonist Ameliorates Septic Immunosuppression through Reducing Immunosuppressive PD‐L1^+^ Neutrophil Subpopulation

**Figure 8:**
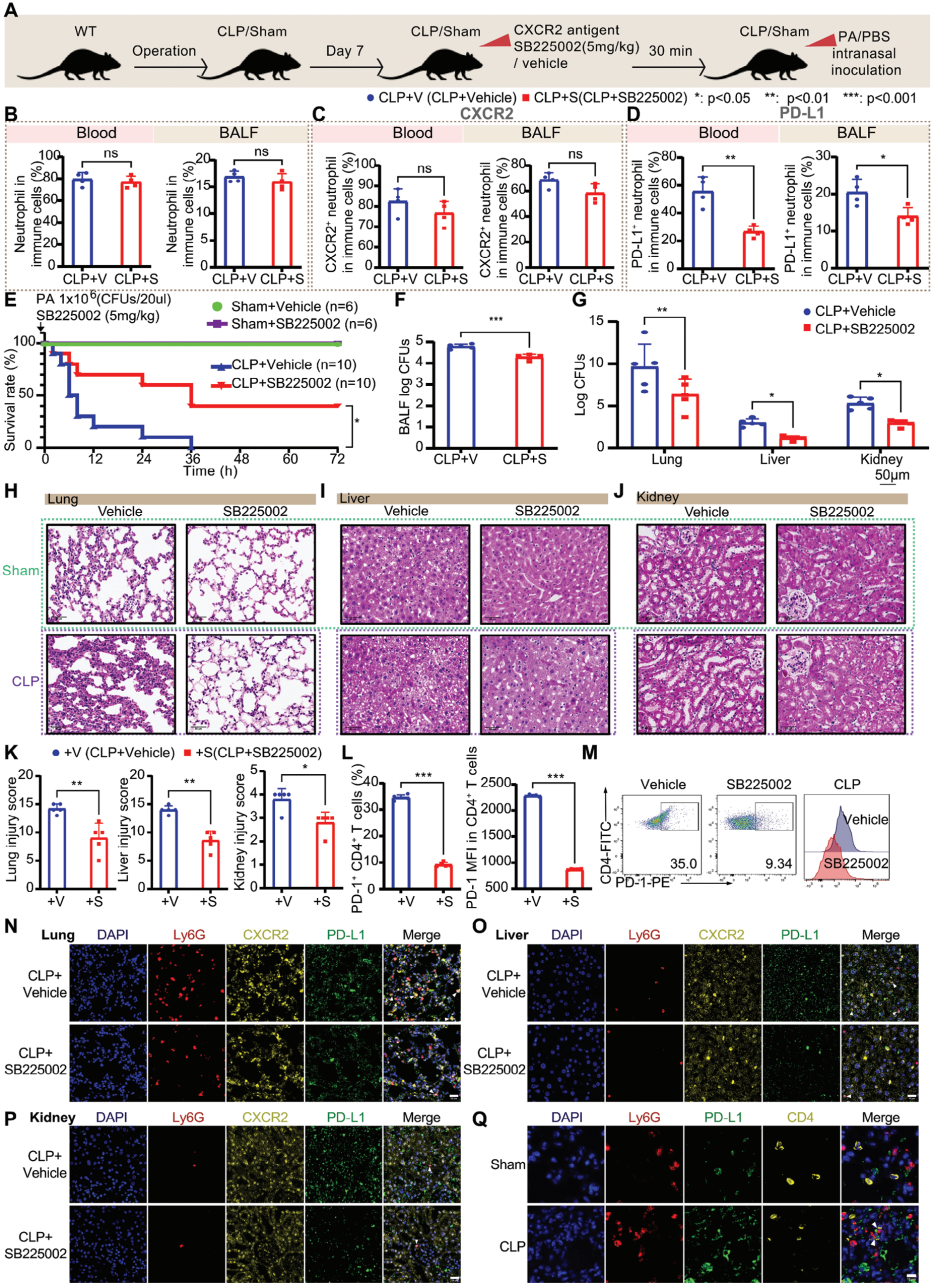
CXCR2 antagonist ameliorates septic immunosuppression by reducing immunosuppressive PD‐L1^+^ neutrophil subpopulation. A) Experimental flow diagram. B) The proportion of neutrophils in peripheral blood and BALF of surviving mice 6 h after PA intranasal inoculation (for each group, *n* = 4). C) The proportion of CXCR2^+^ neutrophils in peripheral blood and BALF of surviving mice 6 h after PA intranasal inoculation (for each group, *n* = 4). D) The proportion of PD‐L1^+^ neutrophils in peripheral blood and BALF of surviving mice 6 h after PA intranasal inoculation (for each group, *n* = 4). E) Survival analysis of CLP mice and Sham mice intranasally inoculated with PA with or without SB225002 administration (Sham+Vehicle group *n* = 6, Sham+SB225002 group *n* = 6, CLP+Vehicle group *n* = 10, CLP+SB225002 group *n* = 10). F) CFUs of bacteria in BALF of surviving mice 6 h after PA intranasal inoculation (for each group, *n* = 5). G) CFUs of bacteria in the lung, liver and kidney of surviving mice 6 h after PA intranasal inoculation (for each group, *n* = 5). H–J) H&E staining of the lung (H), liver (I), kidney (J) injury in surviving mice 6 h after PA intranasal inoculation (scale bar 50 µm). K) Histological scores assessing the lung, liver, kidney injury in surviving mice 6 h after PA intranasal inoculation (for each group, *n* = 5). L,M) The proportion of PD‐1^+^ CD4^+^ T cells and PD‐1 MFI in CD4^+^ T cells in the spleen of surviving mice 6 h after PA intranasal inoculation (for each group, *n* = 5). N–P) Immunofluorescence staining for CXCR2 and PD‐L1 on neutrophils in the lung (N), liver (O), kidney (P) of surviving mice 6 h after PA intranasal inoculation (scale bar 20 µm) (Red, Ly6G; yellow, CXCR2; green, PD‐L1). Q) Immunofluorescence staining for PD‐L1 on neutrophils and CD4^+^ T cells in the lung of CLP mice and Sham mice 7 days post surgery (scale bar 10 µm) (Red, Ly6G; green, PD‐L1; yellow, CD4). ns denotes not significant, * denotes *p *< 0.05, ** denotes *p* < 0.01, *** denotes *p* < 0.001. Student's t test was used for Figure 8B–D,F,K,L. Two‐way ANOVA was used for Figure 8G. Kaplan‐Meier method and Log‐rank test were used for Figure 8E.

To investigate the function of CXCL1/8‐CXCR2 in immunosuppressive stage of sepsis, CLP‐induced sepsis mice were intraperitoneally injected with SB225002, a CXCR2 selective non‐peptide antagonist (**Figure** **8**A).^[^ ^36^ ^]^ Compared with the vehicle group, the proportion and number of neutrophils and macrophages and CXCR2 expression in neutrophils in peripheral blood and BALF of the sepsis mice did not change (Figure 8B,C; Figure S8A–C, Supporting Information). However, the expression of PD‐L1 in neutrophils was significantly downregulated (Figure 8D; Figure S8D, Supporting Information), indicating that antagonizing *CXCR2* restricted the differentiation of immunosuppressive neutrophil in vivo.

More importantly, the application of SB225002 increased the survival rate of CLP mice that received PA intranasal inoculation (Figure 8E). CFUs in BALF of CLP mice after intraperitoneal injection of SB225002 were decreased after the second blow (Figure 8F), and CFUs in lung, liver, and kidney tissues were also decreased (Figure 8G). To observe the changes in different organs in CLP mice after intraperitoneal injection of SB225002, we further evaluated the lung, liver and kidney damage by H&E staining. After SB225002 treatment, organ damage of the lung, liver and kidney was ameliorated in surviving mice (Figure 8H–K). In addition, we also observed that PD‐L1 expression on neutrophils in the lung, liver and kidney in surviving mice was decreased 6 h after PA intranasal inoculation (Figure 8N–P). Given that total neutrophils and CXCR2^+^ neutrophils did not change significantly, SB225002 could accurately reduce the content of PD‐L1^+^ neutrophils (Figure 8N–P). We tested the expression of PD‐1 on CD4^+^ T cells and found that after intraperitoneal injection of SB225002, the content of CD4^+^ T cells expressing PD‐1 was significantly reduced (Figure 8L,M), while direct contact was found between PD‐L1^+^ neutrophils and PD‐1^+^ CD4^+^ T cells in CLP mice (Figure 8Q; Figure S8E, Supporting Information). These results suggest that antagonizing CXCR2 could improve sepsis‐induced immunosuppression through reducing immunosuppressive neutrophil subpopulation.

## 3 Discussion

In sepsis, most patients surviving the initial hyperinflammatory phase entered an immunosuppressive phase.^[^ ^2^ ^]^ The later phase with immunosuppressive state is closely related to adverse long‐term outcomes such as organ failure, during which immune cell dysfunction plays a major role. Bulk and single‐cell transcriptome analyses on peripheral blood and other organs have been utilized to uncover alterations of immune cell composition, phenotypes, and gene signatures driven by sepsis, thus identifying potential biomarkers for the diagnosis or treatment of sepsis.^[^ ^37^, ^38^, ^39^ ^]^ The close connection between sepsis and neutrophils, especially immunosuppressive neutrophil subpopulations, has attracted much attention.^[^ ^17^, ^28^ ^]^ However, it is unclear whether these cells have unique transcriptomic patterns in the pulmonary environment due to limited sequencing data generated from BALF.

In this study, we applied scRNA‐seq to human BALF samples, including those from patients in immunosuppressive phase of sepsis and healthy donors, aiming to dissect the composition changes and heterogeneity of lung immune cells and relevant mechanisms related to the pathogenesis of sepsis. As a result, five neutrophil subpopulations and eight macrophage subpopulations were identified, namely, N00‐N04 and M00‐M07. Compared to healthy donors, the proportion of neutrophils was dramatically increased in BALF and peripheral blood of septic patients. The same trend was observed in CLP septic mouse models. Thus, we hypothesized that neutrophils are crucial in this environment and specifically focused on these subpopulations.

Neutrophils have become an emerging therapeutic target in sepsis due to their important functions.^[^ ^32^ ^]^ Specific subtypes has been identified in peripheral blood by previous studies.^[^ ^17^, ^28^, ^39^, ^40^ ^]^ Besides, several studies have identified an immunosuppressive PD‐L1^+^ neutrophil subpopulation in peripheral blood of sepsis patients.^[^ ^41^, ^42^ ^]^ These studies found that systemic inflammation and cytokines, such as IFN‐γ, influence the PD‐L1^+^ neutrophil subpopulation in peripheral blood. In contrast, PD‐L1^+^ *IL1RN* ^+^ neutrophil subpopulation in BALF is likely regulated by local inflammatory factors such as CXCL1/8, indicating a unique role in local immunosuppression associated with *CXCR2* ^+^ neutrophil subpopulation in the lung environment.

Regardless of specific subtypes identified in peripheral blood by previous studies, our study detected neutrophil subpopulations with different gene signatures in BALF. This might be caused by discrepancies of the origin of cells and the status of disease. The maturation status of *S100A8* ^+^ neutrophils is relatively lower than that of other subtypes, raising the possibility that these cells may further develop into a functional phenotype after entering the lungs. In particular, a subpopulation expressing *CXCR2* (N00) was found to be significantly increased in immunosuppressive septic BALF. *CXCR2* is an important receptor of the CXCL pathway driving the migration of neutrophils across the vascular endothelium to the site of inflammation.^[^ ^43^ ^]^ Previous studies have suggested that the failure of neutrophil migration to the infection site is related to the low expression of *CXCR2*.^[^ ^14^ ^]^ The remarkable enrichment of the *CXCR2^+^* neutrophil subpopulation in our study infers that its infiltration into the lung contributes to the dramatic increase in neutrophils in immunosuppressive septic BALF. Another interesting *CD274* ^+^ *IL1RN* ^+^ subpopulation also displayed a higher proportion in immunosuppressive septic BALF. However, unlike the *CXCR2* ^+^ subpopulation, these cells presented mature and immunosuppressive phenotypes and are very likely to be the major regulators of sepsis‐induced lung injury. On the one hand, studies have found increased expression of PD‐L1 in dendritic cells and epithelial cells in the lungs of septic patients.^[^ ^2^ ^]^ Moreover, conditional deletion of PD‐L1 ameliorated lung injury in a septic mouse model and reduced the quantity of neutrophils in the lungs.^[^ ^23^ ^]^ On the other hand, *IL‐1RN* is an immunomodulatory molecule that normally functions by competing for the binding site of IL‐1R with IL‐1^[^ ^44^ ^]^ and shows anti‐inflammatory effects. *IL‐1RN* polymorphism has been found to be associated with clinical sepsis susceptibility,^[^ ^45^ ^]^ while inhibiting the expression of IL‐1RN induced anti‐sepsis protection in mice.

Immature neutrophils mainly exist and mature in the bone marrow and then migrate to various organs along with the blood, with signature genes during the maturation stage.^[^ ^27^ ^]^ By comparing the neutrophil maturation signature genes and neutrophil subgroups in sepsis blood,^[^ ^17^, ^27^ ^]^ we found that *S100A8* ^+^ neutrophils are likely to be more immature and originate from progenitors in the plasma, and under sepsis conditions, they migrate to the lungs. After entering the lung environment, *S100A8* ^+^ neutrophil progenitors differentiate into intermediate‐state *CXCR2^+^* neutrophils and mature *IL1RN* ^+^PD‐L1^+^ neutrophils. *CD274* ^+^ *IL1RN* ^+^ mature neutrophils are an immunosuppressive neutrophil subset that participates in immunosuppression in the lung environment of patients with sepsis and is a unique cell population in the lung environment, different from the immunosuppressive *IL1R2* ^+^ neutrophil subset in plasma.^[^ ^17^ ^]^ Indeed, trajectory analysis revealed the developmental trajectory from *CXCR2* ^+^ neutrophils to *CD274* ^+^ *IL1RN* ^+^ neutrophils in sepsis BALF, consistent with the maturation state. Together with the results from CLP sepsis mice, we considered *CD274* ^+^ *IL1RN* ^+^ mature neutrophils to be immunosuppressive effector cells in the lung environment during sepsis‐induced immunosuppression, which can transition from intermediate‐state *CXCR2* ^+^ neutrophils.

In addition to neutrophil subpopulations, we also detected subtypes of macrophages and epithelial cells in the BALF of immunosuppressive septic patients and identified their connections with *CXCR2* ^+^ neutrophils. Two macrophage subtypes (M02 and M04) and one epithelial cell subtype (E023) were characterized by *CXCL8/1* positivity, the ligand of *CXCR2*. More importantly, an enhanced *CXCL8/1‐CXCR2* connection was found. *CXCR2* expression in neutrophils was reduced in the blood of severe septic patients, concomitant with a decrease in IL‐8.^[^ ^46^ ^]^ Previous studies have shown that after differentiation from progenitors in the bone marrow, neutrophils shift the chemokine receptor from *CXCR4* to *CXCR2* and are subsequently released into the blood under the chemotaxis of CXCL8 in the blood.^[^ ^32^ ^]^ *CXCL8*/IL‐8 is the most important chemokine in response to the inflammatory response. Previous studies have shown that IL‐8 is increased in patients with lung infection,^[^ ^33^ ^]^ inducing neutrophil recruitment by activating leukocyte chemokine receptors *CXCR1*/*CXCR2*.^[^ ^30^, ^31^ ^]^ A previous study also showed that *CXCL1* can be produced by macrophages and epithelial cells and can recruit and activate neutrophils by binding to its receptor *CXCR2*.^[^ ^47^, ^48^, ^49^, ^50^ ^]^ Our data indicate that the infiltration of *CXCR2* ^+^ neutrophils in the BALF of immunosuppressive septic patients might be attributed to *CXCL8/1* secreted from macrophages/epithelial cells. Furthermore, in vitro stimulation data showed that *CXCL8*‐*IL‐8*/*CXCL1* can induce the expression of PD‐L1 in *CXCR2* ^+^ neutrophils, raising the possibility that CXCL8/1 may also be responsible for the transition from *CXCR2* ^+^ neutrophils to *CD274* ^+^ *IL1RN* ^+^ neutrophils.

In this study, septic mouse models were induced by abdominal infection, whereas the clinical samples included infections from community‐acquired pneumonia, urosepsis, intra‐abdominal sepsis, meningitis, and necrotizing fasciitis. Different infection sites can trigger varying immune responses,^[^ ^51^ ^]^ and this inconsistency may affect the results and their interpretation. Based on our current results, the alterations in the pulmonary immune microenvironment during sepsis‐induced immunosuppression appear consistent across various infection sources. Although CLP‐induced abdominal infection is a standard model for sepsis research, we have not applied sepsis models induced from other infection sites, which is a limitation of this study. In future studies, employing multiple infection models, classifying the immune responses of clinical samples from different infection sites, and further investigating the impact of varying infection sites on sepsis‐induced immunosuppression mechanisms will enhance the validity of the results and provide more targeted guidance for clinical treatment.

In summary, our study dissects neutrophil heterogeneity in BALF by scRNA‐seq analysis and determines that *CXCR2* ^+^ neutrophils are a key subpopulation involved in immunosuppressive phase of sepsis. These results provide critical information for basic research and clinical diagnosis and treatment of sepsis.

## 4 Experimental Section

### Patients

According to the third international consensus definition for sepsis and septic shock^[^ ^52^ ^]^ and the consensus on sepsis‐induced immunosuppression,^[^ ^53^ ^]^ recognized indicators were employed to identify septic immunosuppression: decreased mHLA‐DR levels, reduced lymphocyte counts, and increased Treg proportions. Four patients diagnosed with sepsis and admitted to the ICUs at Zhongshan Hospital, Fudan University for 14 days or more were enrolled in this study for scRNA‐seq. Fifteen immunosuppressive sepsis patients were enrolled for flow cytometric analysis and other experiments. For healthy controls, sixteen individuals who were admitted for evaluation of solitary pulmonary nodules without evidence of pulmonary infection were enrolled. Demographic and clinical characteristics of participants were recorded, including sex, age, body mass index (BMI), final diagnosis, comorbidities, sequential organ failure assessment (SOFA), and acute physiology and chronic health status scoring system II (APACHE II) (Tables S2 and S3, Supporting Information). BALF was collected within 24 h of diagnosis in all patients. This study was approved by the Ethics Committee of Zhongshan Hospital, Fudan University (B2021‐182R), and complied with the ethical standards set out in the Declaration of Helsinki.

### Harvest of BALF

Bronchoalveolar lavage was performed using the standard protocol for septic patients and healthy controls. Briefly, lavage was performed by instilling 20 mL of sterile saline two times (40 mL in total), with an approximate retrieval of 20 mL. Two to three millilitres of the retrieved volume was used for clinical purposes, and the remaining volume was used for scRNA‐seq. The collected BALF was filtered through a 40 µm cell filter (Falcon, 3 523 240) and processed under biosafety S3 conditions within 1 h after collection. Prior to processing, BALF was placed on ice.^[^ ^54^, ^55^ ^]^ Bronchoalveolar lavage in mice was performed using a 20 G intravenous indwelling needle. One milliliter of sterile saline was injected into the lungs of mice, and BALF was recovered after three lavage procedures.^[^ ^56^ ^]^

### Cell Suspension Preparation

The BALF was passed through a 100 µm filter (Falcon, 3 523 260) and centrifuged (400 g, 10 min, 4 °C). Pelleted cells were resuspended in red blood cell lysis buffer (Solarbio, R1010), incubated for 2 min, passed through a 40 µm filter (Falcon, 3 523 240), collected by centrifugation (400 g, 10 min, 4 °C) and resuspended in PBS (BI, 02‐024‐1ACS) containing 0.04% BSA (Sigma, B2064). Cells were manually counted by Trypan blue (Thermo, T10282) and AO‐PI (LUNA, D23001) after each centrifugation (400 g, 10 min, 4 °C) and resuspended. Single cells were processed using Chromium Controller (10X Genomics) according to the manufacturer's protocol.

### scRNA‐seq Library Construction and Sequencing

By using the Chromium Next GEM Single Cell 3′ Kit v3.1 (10x Genomics, 1 000 268) and Chromium Next GEM Chip G Single Cell Kit (10x Genomics, 1 000 120), single‐cell 3′ gene expression profiling was performed. The cell suspension was loaded onto the Chromium single‐cell controller (10x Genomics) to generate single‐cell gel beads in the emulsion according to the manufacturer's protocol. Captured cells were lysed, and the released RNA was barcoded through reverse transcription in individual GEMs. Cell‐barcoded 3′ gene expression libraries were sequenced on the Illumina NovaSeq6000 system by Shanghai Biochip Co., Ltd. (Shanghai, China). The raw data were uploaded to the National Omics Data Encyclopedia (NODE) database. The accession number for the processed data from human samples in this paper is https://www.biosino.org/node/project/detail/OEP003526.

### scRNA‐seq Data Analysis**—**Publicly Available Data

In addition to the BALF data from the control H1, raw scRNA‐seq data from BALF of three additional controls H2‐4 were retrieved from the Gene Expression Omnibus (GEO) database, accession code GSE145926.^[^ ^18^ ^]^ These samples were processed using the same workflow as the dataset and were analyzed together with the dataset to ensure rigorous cross‐comparison and consistency.

### scRNA‐seq Data Analysis**—**Raw Data Processing

CellRanger‐v6.1.1 was used to process raw scRNA‐seq data.^[^ ^57^ ^]^ Briefly, the raw fastq data were mapped to the reference genome (GRCh38‐2020‐A) to generate gene‐arcode matrices of gene expression. Following the CellRanger technical note (CG000148), for the P2 and P3 data parameter, force cells were set to 1500–1600. Other samples were processed with CellRanger default parameters.

### Unsupervised Clustering Analysis and Cell Cluster Identification

All downstream analyses were conducted in R 4.1.1. Preprocessing analysis was performed using the R package Seurat v4.1.1.^[^ ^58^, ^59^, ^60^, ^61^ ^]^ The pipeline described in article was generally followed,^[^ ^18^ ^]^ with the modification of not applying an nUMI cutoff. This adjustment was made to retain a broader range of neutrophils, in accordance with recommendations from 10x Genomics (tutorials/cr‐tutorial‐neutrophils) and considerations noted in this article.^[^ ^62^ ^]^ Low‐quality cells were filtered based on high mitochondrial gene percentages (>10%) or an anomalous number of detected features (>6000 or <200). Log normalization was performed with default parameters. Highly variable features were detected using the “vst” method with default settings.

Subsequently, each sample was integrated for downstream analysis. To correct batch effects, the dataset was split based on the source identities using the SplitObject function. Each subset was normalized using NormalizeData, and highly variable features were identified with FindVariableFeatures, using the “vst” selection method and nfeatures set to 2000. Integration features were then selected using SelectIntegrationFeatures with default parameters. Integration anchors were identified with FindIntegrationAnchors using the selected features. The data was finally integrated using IntegrateData with the identified anchors. Dimensionality reduction was conducted using principal component analysis (PCA) and uniform manifold approximation and projection (UMAP) to visualize the integrated data. Cell clusters were identified using FindNeighbors and FindClusters, employing the default Louvain algorithm. The resolution parameter was adjusted to 1.2 based on parameter tuning to optimize clustering outcomes.

### Cell Cluster Annotation

Both novel markers and the R package SingleR (HumanPrimaryCellAtlasData) and scMRMA were used to annotate cell clusters (on the Seurat “RNA” assay).^[^ ^63^, ^64^ ^]^ Briefly, annotation was performed for the integrated object and for each sample separately for comparison. For SingleR, the HumanPrimaryCellAtlasData (hpca.se) reference was used to infer cell types. The annotation of the integrated object was then corrected based on SingleR annotation and expression of novel markers, during which doublets were identified manually based on annotation and expression of novel markers.

### Differential Expression Analysis and Enrichment Analysis

The R packages Seurat and ggplot2 were used to identify differentially expressed genes and visualize the analysis results.^[^ ^58^, ^59^, ^60^, ^61^ ^]^ The FindAllMarkers function was used to identify cluster‐specific genes among different cell populations with min.pct = 0.25 and logfc.threshold = 0.5. The FindMarkers function was used to identify differentially expressed genes between two subpopulations or between patient and healthy cell populations, with the parameters logfc.threshold = log(1.2), min.pct = 0.2, and only.pos = FALSE. The Wilcoxon rank‐sum test, followed by Bonferroni correction, was applied in both analyses.

The R package clusterProfiler was used in enrichment analysis.^[^ ^65^, ^66^ ^]^ All enrichment analyses were based on the Gene Ontology (GO) and Kyoto Encyclopedia of Genes and Genomes (KEGG) databases. Enrichment results were adjusted by incorporating gene p‐values as weights to assess the significance of GO terms more accurately. This adjustment involved multiplying the p‐values of each GO term by the mean of the adjusted p‐values of the genes associated with that term, providing a weighted analysis. Enrichment results were shown in dot plots and bar charts using the R package ggplot2.

### Identification of Specific Subpopulations of Neutrophils and Macrophages

Neutrophils and macrophages were reclustered separately to reveal the cell heterogeneity within these cell groups. Specifically, resolution *n* = 0.6 was used for neutrophil clusters and 0.8 for macrophages. To identify the cell composition changes in neutrophils and macrophages, on the one hand, the absolute proportion of a subpopulation was calculated among all cells of each sample. Then, the absolute proportion of all patients was compared to that of controls (Student's t test). On the other hand, for neutrophils, the relative proportion of a subpopulation was calculated among all neutrophils from each sample, after which the average relative proportion was displayed in a pie chart using the R package ggplot2.

### Cell‒Cell Communication Analysis and Significant Ligand‒Receptor Pair Identification

Important interactions were identified based on the integrated differential analysis results and the cellular communication changes between patients and controls. CellChat‐v1.1.3 was applied with default parameters to perform cellular communication analysis.^[^ ^67^ ^]^ Single‐cell data from patients and controls were analyzed independently; therefore, two CellChat objects were generated. A network using six cell types was built. Overexpressed genes and interactions were identified by the identifyOverExpressedGenes and identifyOverExpressedInteractions functions (*P* < 0.05). Then, computeCommunProb, computeCommunProbPathway and netAnalysis_computeCentrality were used to analyze the importance of interaction in patients and healthy controls separately. After network analysis, the netVisual function was used to visualize enriched signaling pathways.

### Developmental Trajectory Inference

Three different methods, including Velocyto‐v0.17.17,^[^ ^68^ ^]^ Slingshot‐v2.2.1,^[^ ^69^ ^]^ and Monocle3‐v1.0.0,^[^ ^70^, ^71^, ^72^, ^73^ ^]^ were used to infer the developmental trajectory of neutrophil subpopulations. Velocyto‐v0.17.17 was applied to compute the RNA velocities of single cells with default parameters for each sample separately. Specifically, velocyto run10x was used, with the CellRanger count data as input, to compute the velocity loom file including spliced, unspliced and ambiguous information of each cell. Then, the loom files were processed based on the velocyto.R‐v0.6 package and SeuratWrappers‐v0.3.0 package. The read.loom.matrices function from velocyto.R package was used to read the loom matrices. The RunVelocity function from the SeuratWrappers package was applied to run velocity from the spliced and unspliced assays. The velocities were visualized in the precomputed UMAP embedding using the show.velocity.on.embedding.cor function. Slingshot‐v2.2.1 and Monocle3‐v1.0.0 were applied to infer the sepsis‐specific trajectory of neutrophils, the composition of which dramatically changed in sepsis BALF. Slingshot was performed based on reduced dimensionality data via both PCA and UMAP, with N01 healthy cells as the initial cluster. Monocle3 was performed based on Seurat object and reduced dimensionality data via both PCA and UMAP, with healthy neutrophil cells as the root cluster.

### Mice

C57BL/6J male mice aged 8–10 weeks weighing 20–25 g were obtained from Shanghai Laboratory Animal Research Center (Shanghai, China). All animals were maintained by the Department of Laboratory Animal Science of Fudan University under a 12–12 h light‐dark cycle and specific pathogen‐free conditions. All animal experiments were conducted in accordance with the guidelines of the Animal Review Committee of Zhongshan Hospital, Fudan University (2020‐119).

### Sepsis Model

In this study, a sepsis model was established by cecal ligation and puncture (CLP).^[^ ^74^ ^]^ Briefly, a median abdominal incision was made in mice after anesthesia and skin preparation. The exposed cecum was ligated from the distal cecum to the median of the ileocecal valve and punctured once with a 22G needle from the mesentery toward the antimesenteric direction, and a small amount of feces was extruded. The muscle layer and skin layer were sutured sequentially after retraction of the cecum. Mice in the sham‐operated group received the same protocol without cecal ligation and puncture. It was shown that surviving mice exhibit persistent immunosuppression 8 days after CLP modeling.^[^ ^20^ ^]^ Therefore, 1/3/7 days after CLP operation were used as the main observation time points for sepsis immunosuppression in this study.

In specific experiments, sham‐operated and septic mice were intraperitoneally injected with SB225002, a selective CXCR2 nonpeptide antagonist (5 mg kg^−1^, Med Chem Express, HY‐16711) on day 7 postoperatively.^[^ ^36^ ^]^ Each mouse anesthetized with 3% isoflurane in oxygen was intranasally inoculated with 20 µL of *Pseudomonas aeruginosa* containing 1 × 10^6^ CFUs of bacteria 30 min later to establish a second‐strike model. Bacterial preparation was performed as previously described.^[^ ^20^ ^]^ Peripheral blood, BALF, lung, liver and kidney samples were collected for histopathological, flow cytometric analysis and other experiments (Table S4, Supporting Information).

### Flow Cytometry

Single‐cell suspensions of mouse BALF were prepared. Peripheral blood samples from mice were suspended in cell staining buffer (BioLegend, 420 201) after erythrocyte lysis (BioLegend, 420 301) and centrifugation and incubated with anti‐CD16/32 antibody (BioLegend, 101 320) at room temperature for 10 min to block nonspecific binding sites. Depending on the experimental purpose, cells were incubated with Fixable Viability Stain 510 (BD Biosciences, 564 406), PerCP/Cy5.5‐conjugated CD45 (BioLegend, 103 132/304 028), FITC‐conjugated CD11b (BioLegend, 101 206), PE‐conjugated CXCR2 (BioLegend, 149 304/320 706), APC‐conjugated Ly‐6G (BioLegend, 127 614), BV421‐conjugated PD‐L1 (BD Biosciences, 564 716), APC‐conjugated PD‐L1 (BioLegend, 329 708), BV421‐conjugated CD15 (BioLegend, 310 824), APC‐Cy7‐conjugated CD3 (BioLegend, 100 222/317 342), PE‐conjugated CD4 (BioLegend, 100 408/300 508), PerCP/Cy5.5‐conjugated CD8 (BioLegend, 100 734), BV421‐conjugated CD25 (BioLegend, 102 034), PE‐conjugated CD25 (BioLegend, 302 606), AF647‐conjugated FOXP3 (BioLegend, 320 014), BV421‐conjugated CD127 (BioLegend, 351 310), PE‐Cy7‐conjugated PD‐1 (BioLegend, 329 918), PE‐conjugated PD‐1 (BioLegend, 329 905), PerCP/Cy5.5‐conjugated CD69 (BioLegend, 310 926), PE‐conjugated IFN‐γ (BioLegend, 383 303), PE‐conjugated MCP‐1 (BioLegend, 502 604), APC‐conjugated ARG1 (BioLegend, 369 705) and PerCP/Cy5.5‐conjugated HLA‐DR (BioLegend, 307 629) for the corresponding time. According to the experimental purposes, MMP9 (Absin, abs119896) or IL1RN (Absin, abs115965) antibodies were added to dilutions in an appropriate proportion and resuspended in AF488‐conjugated secondary antibody diluent (incubation at 4 °C, antibody dosage and incubation time were subject to product instructions). Stained cells were analyzed using FACSVerse flow cytometry, and data files were analyzed using FlowJo 10.0.7 software.

### Bacterial CFUs

BALF, lung, liver, and kidney samples were obtained to determine bacterial numbers. Tissues underwent homogenization initially. Each sample was diluted at 100 times, and then 10 µl of diluted sample was plated on LB‐Agar plates. Experiments were performed by triplicates for each sample, and the CFUs for each sample was calculated by an average of CFU counts from three plates. Plate cultures were incubated overnight at 37 °C, and colony counts were then determined.

### H&E Staining

Lung, liver, and kidney tissues were collected, washed with PBS, fixed with 4% paraformaldehyde (PFA), gradient dehydrated with ethanol and embedded in paraffin to make 4 µm sections. Paraffin sections were dewaxed and then stained with hematoxylin and eosin and finally analyzed under a light microscope (Carl Zeiss, Jena, Germany) for histopathological changes.

The histological lung injury score was calculated based on the sum of scores for alveolar edema, alveolar hemorrhage, interstitial thickening, and neutrophil infiltration.^[^ ^52^ ^]^ Each histological feature was evaluated on a scale of 0 to 4: 0 indicates normal (no injury), 1 indicates minimal (injury to 25% of the field), 2 indicates mild (injury between 25% and 50% of the field), 3 indicates moderate (injury between 50% and 75% of the field), and 4 indicates severe (injury over 75% of the field).

The histological liver injury was scored according to necrosis, sinus congestion and edema, lipid vacuoles, and infiltration of erythrocytes and inflammatory cells.^[^ ^75^ ^]^ Each histological character was assessed on a scale of 0 to 4: 0 indicates normal (no injury), 1, 2, 3, and 4 indicate minimal (<25% liver involvement), mild (25–50% liver involvement), significant (50–75% liver involvement), and severe (>75% liver involvement) injury, respectively.

The histological kidney injury score was evaluated by the percentage of damaged renal tubules, as indicated by tubular lysis, dilation, disruption, and cast formation.^[^ ^76^ ^]^ Tissue damage was scored on a scale of 0 to 4, with 0, 1, 2, 3, and 4 corresponding to 0%, <25%, 25–50%, 50–75%, and >75% of damaged renal tubules, respectively.

### Immunofluorescence

Lung, liver, kidney, and spleen tissues collected from mice were fixed with 4% PFA (Servicebio, Cat # G1101) for 15 min and blocked with immunostaining blocking solution (Beyotime, Cat # P0260) for 1 h. They were then incubated with Ly‐6G (Proteintech, 65078‐1‐Ig), CXCR2 (Proteintech, 19538‐1‐AP), PD‐L1 (Proteintech, 66248‐1‐Ig), CD4 (Proteintech, 67786‐1‐Ig) and PD‐1 (Proteintech, 66220‐1‐Ig) antibodies at 4 °C overnight and incubated with relevant secondary antibodies for 1 h at room temperature. Total DNA were stained with 4′,6‐diamidino‐2‐phenylindole (DAPI) (SouthernBiotech, Cat # 0100–20). Immunofluorescence was visualized under an Olympus microscope and data were collected with ImageJ software.

### Isolation and Activation of Neutrophils

Blood samples and BALF samples were collected from 3 healthy volunteers for neutrophil isolation. Monocytes were removed from whole blood or BALF by density gradient centrifugation with Ficoll‐Paque (Cytiva, 17 544 203). Peripheral blood neutrophils were finally obtained after dextran sedimentation and erythrocyte lysis. Cell viability was >93% after isolation. The purity of the isolated neutrophils was routinely >90%. Neutrophils were resuspended in RPMI 1640 medium (1 × 10^6^ mL^−1^) containing 10% fetal bovine serum, 100 U mL^−1^ penicillin and 100 mg mL^−1^ streptomycin. Freshly isolated neutrophils were cultured in six‐well cell culture plates and stimulated with recombinant human IL‐8 (250–1000 ng mL^−1^, PeproTech, 200–08 m) and recombinant human CXCL1 (10–100 ng mL^−1^, MedChemExpress, HY‐P70508) for 1 h.^[^ ^77^, ^78^ ^]^ Reparixin (1 mm, MedChemExpress, HY‐15251) was used for IL‐8 and CXCL1 inhibition.

### Neutrophil‐CD4^+^ T Cell Co‐Culture

CD4^+^ T cells were isolated from peripheral blood of healthy volunteers using the Whole Blood CD4 T Cell Isolation Kit (Miltenyi, 130‐098‐195).^[^ ^17^, ^79^ ^]^ The obtained CD4^+^ T cells were pre‐labeled using 5 µm CFSE (Biolegend, 423 801). CD4^+^ T cells were co‐cultured with PD‐L1^+^/PD‐L1^−^ neutrophils or CXCR2^+^/CXCR2^−^ neutrophils with or without CXCL1 treatment in 48‐well plates (2 × 10^5^ cells per well) at 37 °C in a 5% CO_2_ cell culture incubator at a 4:1 neutrophil to T cell ratio in complete medium supplemented with anti‐CD3 (5 µg mL^−1^, BioXCell, BE0001‐1), anti‐CD28 (2 µg mL^−1^, BioXCell, BE0015‐1), and recombinant human IL‐2 (200 U mL^−1^, PeproTech, 200–02). After 72 h of co‐culture, cells were harvested for flow cytometry.

### Enzyme‐Linked Immunosorbent Assay (ELISA)

Human and mouse peripheral blood was left at room temperature for 20 min and then centrifuged at 3000 rpm for 20 min to collect the supernatant, yielding the peripheral blood plasma. The supernatant of neutrophil‐CD4^+^ T cell co‐cultured medium in vitro was collected after centrifugation at 2000 rpm for 5 min and stored at ‐80 °C for further analysis. The concentrations of mHLA‐DR in peripheral blood plasma and IFN‐γ in cell culture supernatant were determined using ELISA kit (MULTISCIENCES, China) following the manufacturer's instructions.

### Western Blotting

Peripheral blood neutrophils stimulated by recombinant human IL‐8 were collected, and the supernatant was obtained after lysis on ice and centrifugation. Protein concentration was determined using BCA protein assay reagent (Thermo Fisher Scientific, 23 225). Protein samples (30 µg) were separated by 10% SDS‒PAGE and transferred to PVDF membranes (Millipore, ISEQ00010). Membranes were blocked with 5% nonfat milk, probed with the primary antibody against PD‐L1 (Cell Signaling Technology, 13 684) at 4 °C overnight, and then incubated with HRP‐conjugated anti‐rabbit secondary antibody at room temperature for 1 h. The bands were detected using an ECL chemiluminescence kit (Millipore, WBKLS0500) and ImageQuant LAS 4000 imager (GE Healthcare). The intensity of bands was measured using ImageJ 1.8.0 software. The relative expression of PD‐L1 was quantified as the ratio of the intensity of PD‐L1 to GAPDH in the same sample.

### Statistical Analysis

Data from the replication experiments in this study were expressed as the mean ± standard deviation, and all statistical analyses were performed using SPSS 20.0 and GraphPad Prism 9.3 software. All experiments were independently repeated at least three times. The means of two independent samples were compared by Student's t test. The means of multiple independent samples were compared using one‐way ANOVA. Two‐way ANOVA was performed to analyze time and operation using Turkey HSD test. Survival analysis was calculated by the Kaplan‒Meier method and compared with the log‐rank test. A P value<0.05 was considered statistically significant.

### Ethical Declaration

This study was approved by the Ethics Committee of Zhongshan Hospital, Fudan University (B2021‐182R), and complied with the ethical standards set out in the Declaration of Helsinki. Written informed consent was obtained from patients or their relatives for this study. All animal experiments were conducted in accordance with the guidelines of the Animal Review Committee of Zhongshan Hospital, Fudan University (2020‐119).

## Conflict of Interest

The authors declare no conflict of interest.

## Author Contributions

R.S., Y.J., and G.L. contributed equally to this work. R.S., W.K.C., and Z.Z.W. designed the study; R.S., Y.J., G.L.L., S.J.G., H.S., X.Y.W., J.H.G., H.W., K.M., X.N., R.B.A., and W.J.S. performed the experiments; R.S., J.Z., J.W., C.H.M., W.K.C., and Z.Z.W. analyzed the data; R.S., Y.J., W.K.C., and Z.Z.W. wrote the paper; W.K.C. and Z.Z.W. revised the manuscript; all authors gave final approval of the version to be published.

## Supporting information



Supporting Information

## Data Availability

The data that support the findings of this study are available from the corresponding author upon reasonable request.
