# Supplementary material for: Single‐Cell Landscape of Bronchoalveolar Lavage Fluid Identifies Specific Neutrophils during Septic Immunosuppression
Source: Adv Sci (Weinh). 2025 Jan 30;12(11):2406218. doi: 10.1002/advs.202406218 (PMC11923989; doi:10.1002/advs.202406218)
Supplement: Supplementary file 1 — Supporting Information [file ADVS-12-2406218-s001.docx]

**Supplementary figures:**

**Figure S1. Annotation of different cell types and overview of sepsis mouse model.** (A) The proportion of Tregs in peripheral blood of patients and controls (for each group, n=15). (B) Lymphocyte counts in peripheral blood of patients and controls (for each group, n=15). (C) Levels of mHLA-DR in peripheral blood plasma of patients and controls (for each group, n=15). (D) UMAP presentation of different samples. (E) UMAP presentation of different clusters. (F) Composition of patient and healthy cells in each cluster. (G) Cell type composition in patient, control and public control (Liao et al., 2019) samples. (H) Novel markers of different cell types. (I) Relative weight changes in CLP mice and Sham mice compared to baseline (for each group, n=5). (J) Survival analysis of CLP mice and Sham mice within 7 days after surgery (Sham group n=5, CLP group n=10). (K) CFUs of bacteria in BALF of CLP mice and Sham mice 7 days after surgery (for each group, n=5). (L) Total cell counts in extracted BALF of CLP mice and Sham mice (for each group, n=5). (M-O) The proportions of CD4^+^ T cells, CD8^+^ T cells, and Tregs in peripheral blood of CLP mice and Sham mice on day 7 (for each group, n=5). (P) Lymphocyte counts in peripheral blood of CLP mice and Sham mice 7 days after surgery (for each group, n=5). (Q) The level of mHLA-DR in peripheral blood plasma. * denotes p<0.05, ** denotes p<0.01, *** denotes p<0.001. Student’s t test was used for Figure S1A-C, 1K-Q. Two-way ANOVA was used for Figure S1I. Kaplan-Meier method and Log-rank test were used for Figure S1J.


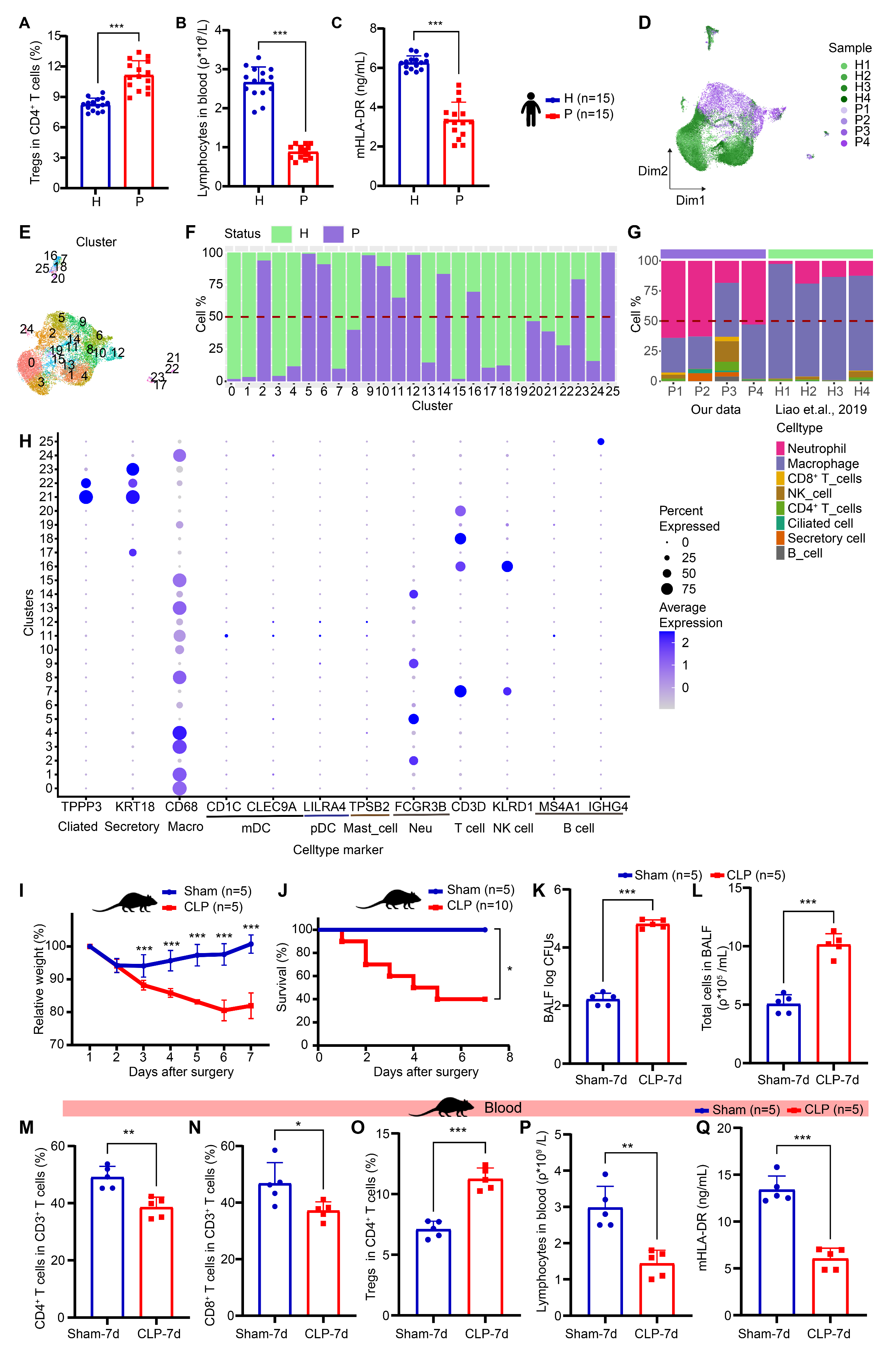


**Figure S2. Differential expression between sepsis patients and healthy controls.** (A) Number of differentially expressed genes (DEGs) between sepsis patients and healthy controls in different cell types. Red bar denotes DEGs with increased expression in patients, and green bar denotes DEGs with decreased expression in patients. (B) Top GO terms enriched by neutrophil DEGs. The red bar denotes the top 20 enriched GO terms of DEGs with increased expression in patients, and the green bar denotes the top 20 enriched GO terms of DEGs with decreased expression in patients. (C) DEGs between sepsis patients and healthy controls in macrophages. Red dots denote DEGs with increased expression in patients, and green dots denote DEGs with decreased expression in patients.


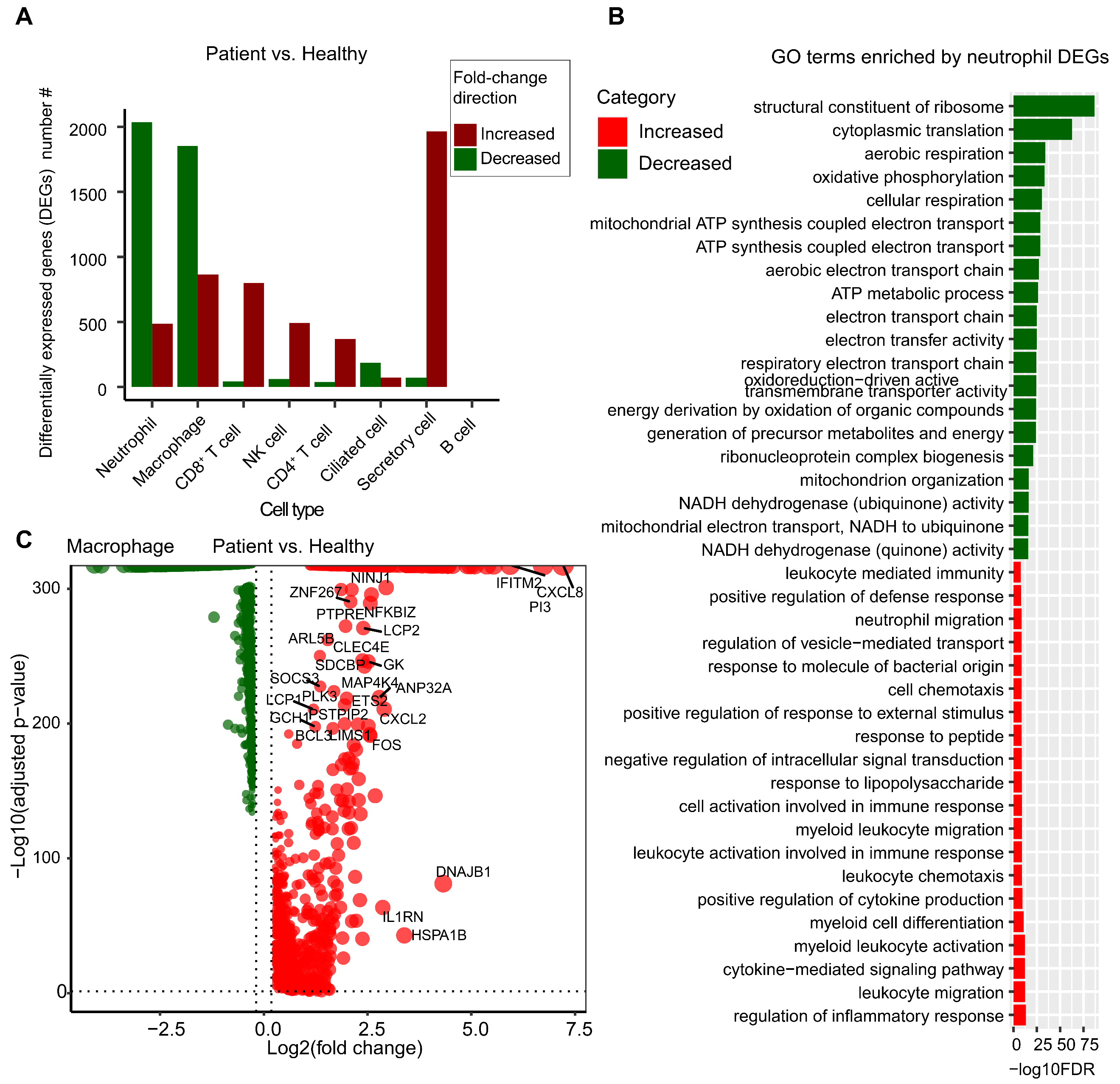


**Figure S3. Annotation and differential expression between septic patients and healthy controls in the neutrophil subgroup.** (A) Maturation heterogeneity in our neutrophil subgroup compared to the public neutrophil maturation dataset (Xie et al., 2020) (Fisher’s exact test, one-sided, p<0.05, OR>1). (B) Enrichment of our neutrophil subgroup in the public blood neutrophil dataset (Hong et al., 2022) (Fisher’s exact test, one-sided, p<0.05, OR>1). (C) Monocle pseudotime of the neutrophil subgroup integrating sepsis blood and BALF data. Neutrophil progenitors from blood were set as roots. Colors denote different cell groups. (D) Top 10 differentially expressed genes (DEGs) in neutrophil clusters. Red characters denote the specific DEG (*IL1RN*) of N02. (E) Shared enriched GO terms of downregulated DEGs in patients (in green color, >= 2 neutrophil clusters) and of upregulated DEGs in patients (in red color, >= 2 neutrophil clusters). Upregulated/downregulated GO terms of DEGs are shown in pink/green. (F) Shared enriched KEGG pathways of downregulated DE genes in patients (in green color, >= 1 neutrophil cluster) and of upregulated KEGG pathway genes in patients (in red color, >= 1 neutrophil cluster). Upregulated/downregulated KEGG pathways are shown in pink/green.


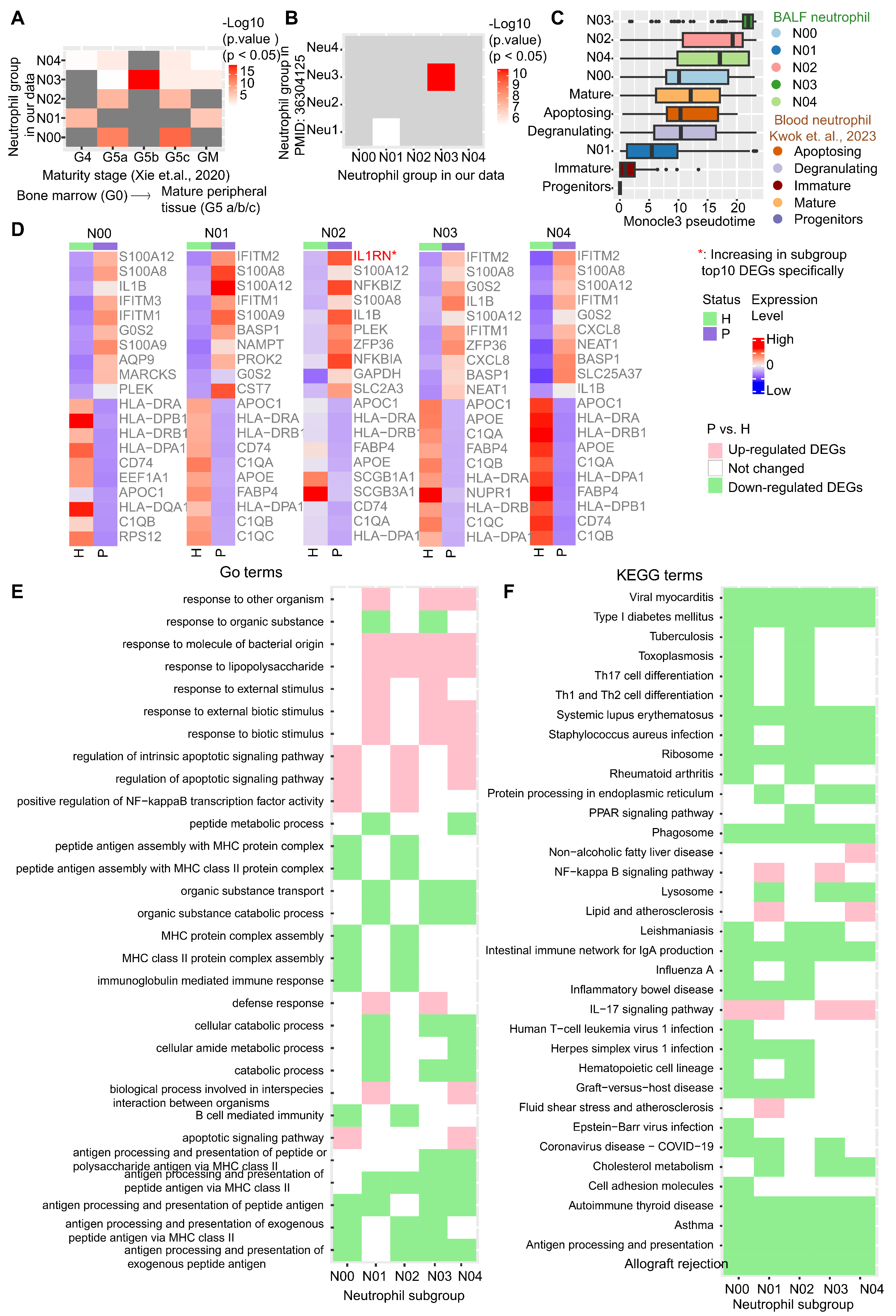


**Figure S4. Increased *CXCR2***^+^ **and IL1RN^+^CD274^+^ neutrophil subgroups in sepsis patients.** (A-B) Representative flow cytometry plots for the proportion of CXCR2^+^ neutrophils and CXCR2 MFI in peripheral blood and BALF of patients and controls (corresponding to Figure 5C-D). (C-D) Representative flow cytometry plots for the proportion of PD-L1^+^ neutrophils and PD-L1 MFI in peripheral blood and BALF of patients and controls (corresponding to Figure 5E-F). (E) *IL1B, IL1RN, and IL1R2* expression in different cell types. (F) *IL1* signaling pathway change in patients (using CellChat). The purple bar denotes patient cell signaling importance. Red characters denote enhanced importance/strength of the signal in patients compared to healthy controls. (G-H) Enhanced *IL1B-IL1R2* cell interaction analysis in macrophages and neutrophils in patients compared to healthy controls (using CellChat). Red characters denote enhanced importance/strength of the signal in patients compared to healthy controls.


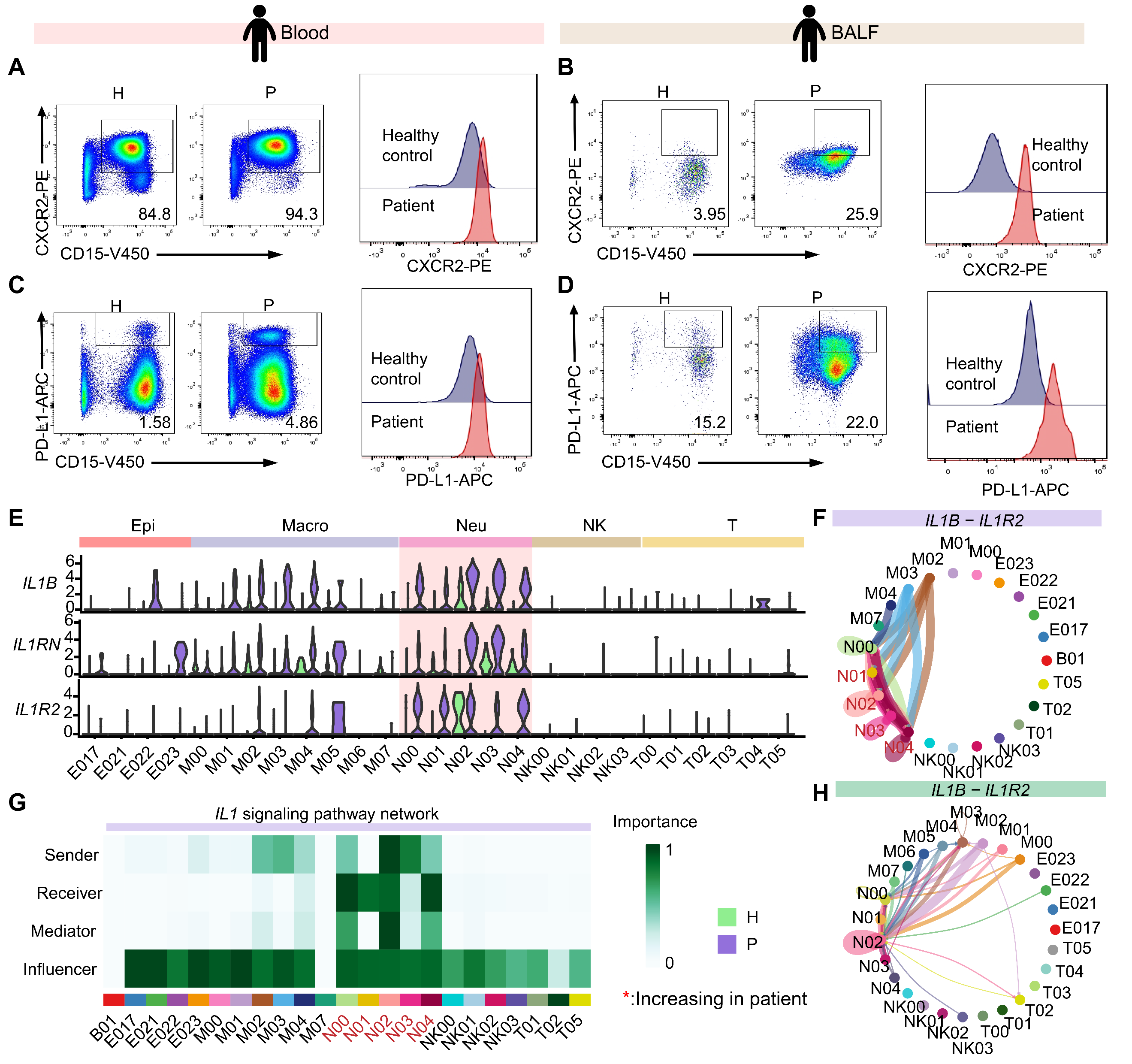


**Figure S5. Regulation of *CXCR2*^+^ and IL1RN^+^CD274^+^ neutrophil subgroups.** (A) RNA velocity analysis of neutrophil clusters in sepsis patient (P2, P4) and healthy (H2) samples. (B) *CXCL* signaling pathway change in patients (using CellChat). The purple bar denotes patient cell signaling importance. Red characters denote increased cell signal importance in patients. (C) Enhanced *CXCL8-CXCR2 and CXCL1/CXCR2* cell interaction analysis in macrophages and neutrophils in patients compared to healthy controls (using CellChat). The purple bar denotes patient cell signaling, and the green bar denotes control cell signaling.


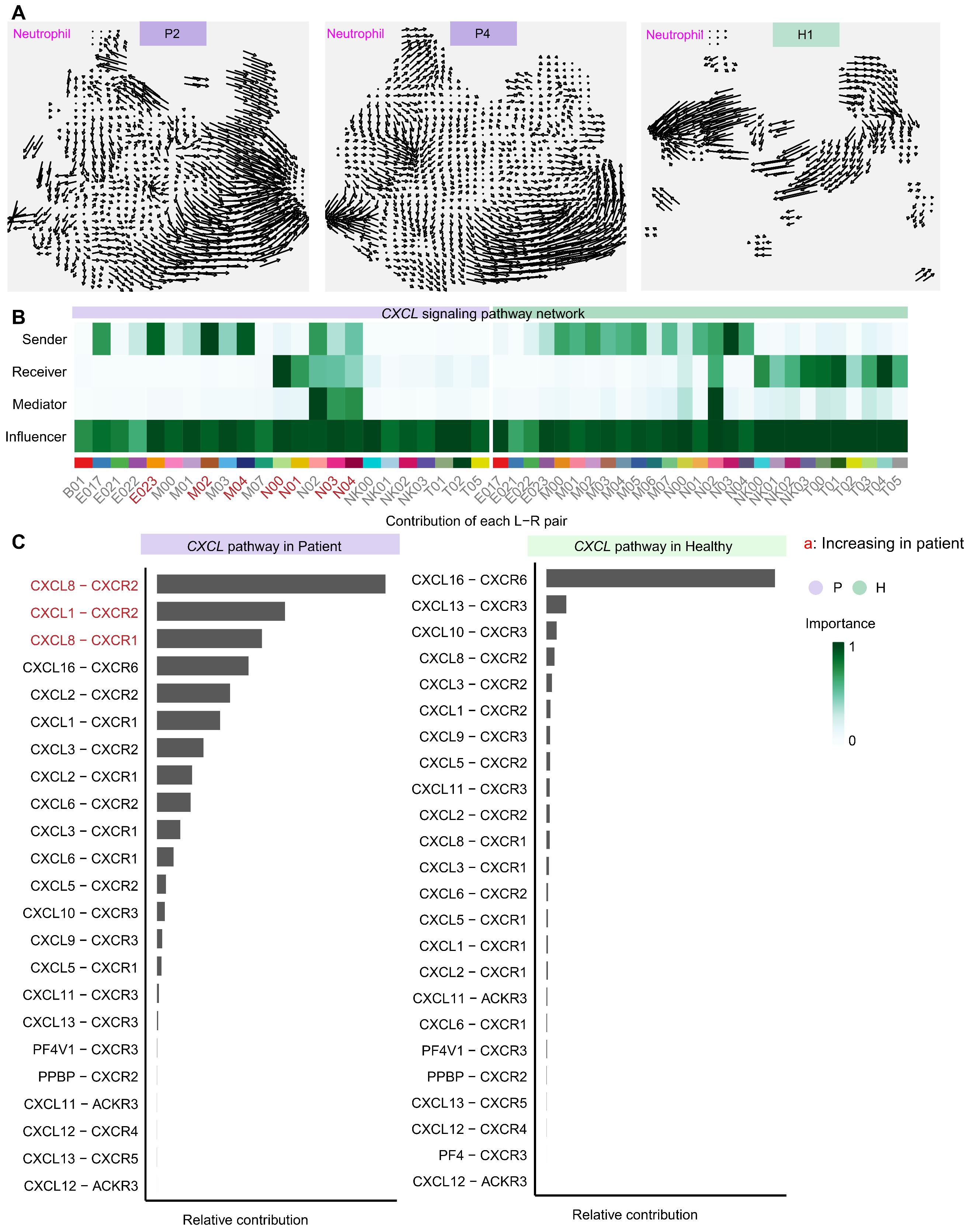


**Figure S6. Heterogeneity of macrophages and *CXCL8*^+^ macrophage subpopulations in sepsis.** (A) UMAP presentation of macrophage subgroups M00-M07 and their specific markers. (B) UMAP presentation of patient (P) and healthy (H) macrophages. (C) Top signature genes in macrophage subgroup M00-M07. (D) Proportion of macrophage clusters in control (green bar) and patient BALF (purple bar); comparison of proportion in H and P (Student’s t test). (E) Expression of macrophage pro-inflammatory-M1 and anti-inflammatory-M2 type markers in subgroups. (F) Top 10 DEGs between patients and healthy controls in subgroups. *CXCL8/CXCL1* are marked in red.


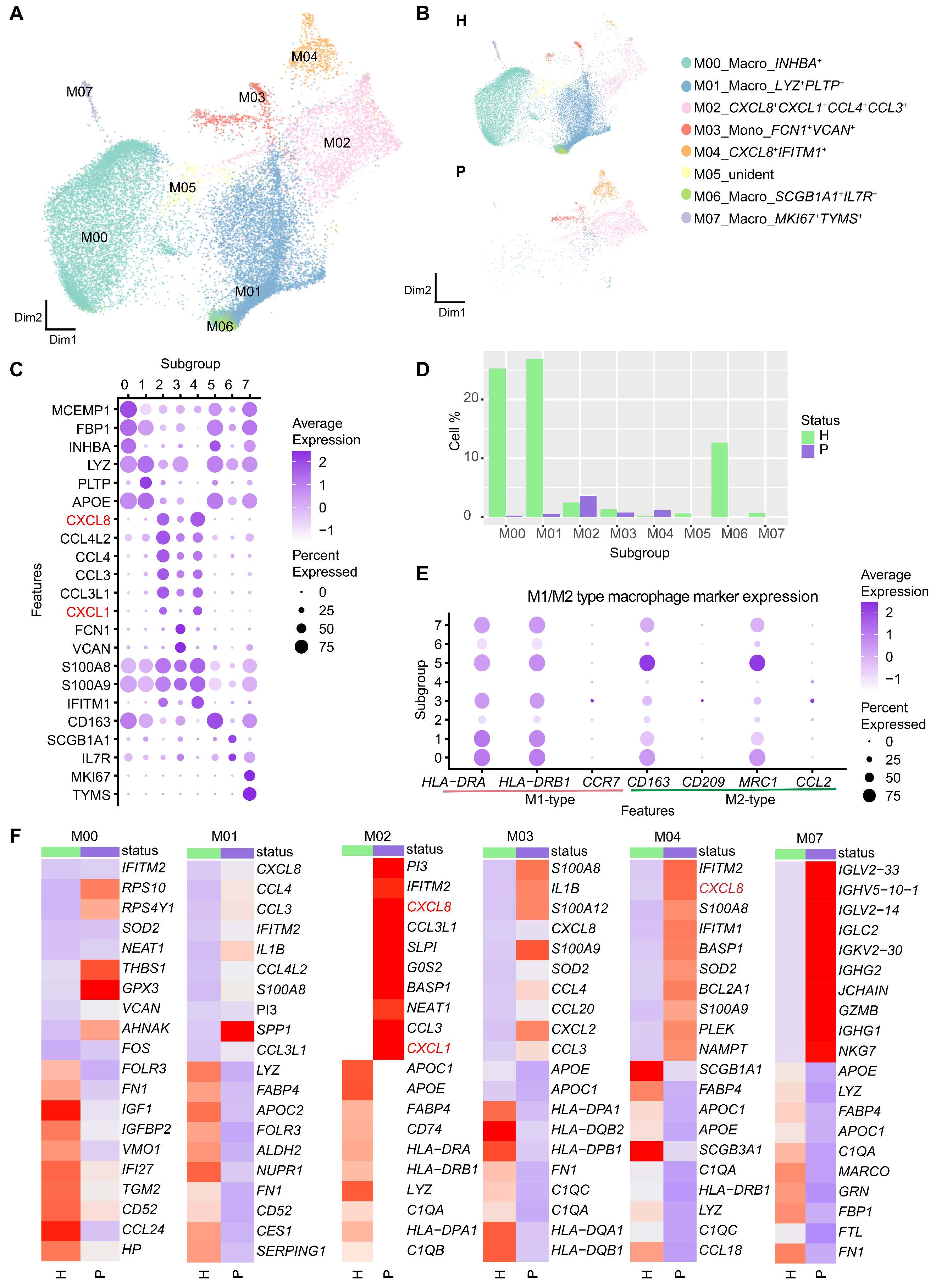


**Figure S7. Cell heterogeneity between sepsis patients and healthy controls in T cells, epithelial cells and NK cells.** (A-B) UMAP presentation of T-cell clusters T00-N05 among all samples and patients and healthy controls (A) and specific marker gene expression (B). (C-D) UMAP presentation of epithelial cell clusters E17, E21, E22, E23 among all samples and patients and healthy controls (C) and specific marker gene expression (D). (E-F) UMAP presentation of NK cell clusters NK00-NK03 among all samples and patients and healthy controls (E) and specific marker gene expression (F).


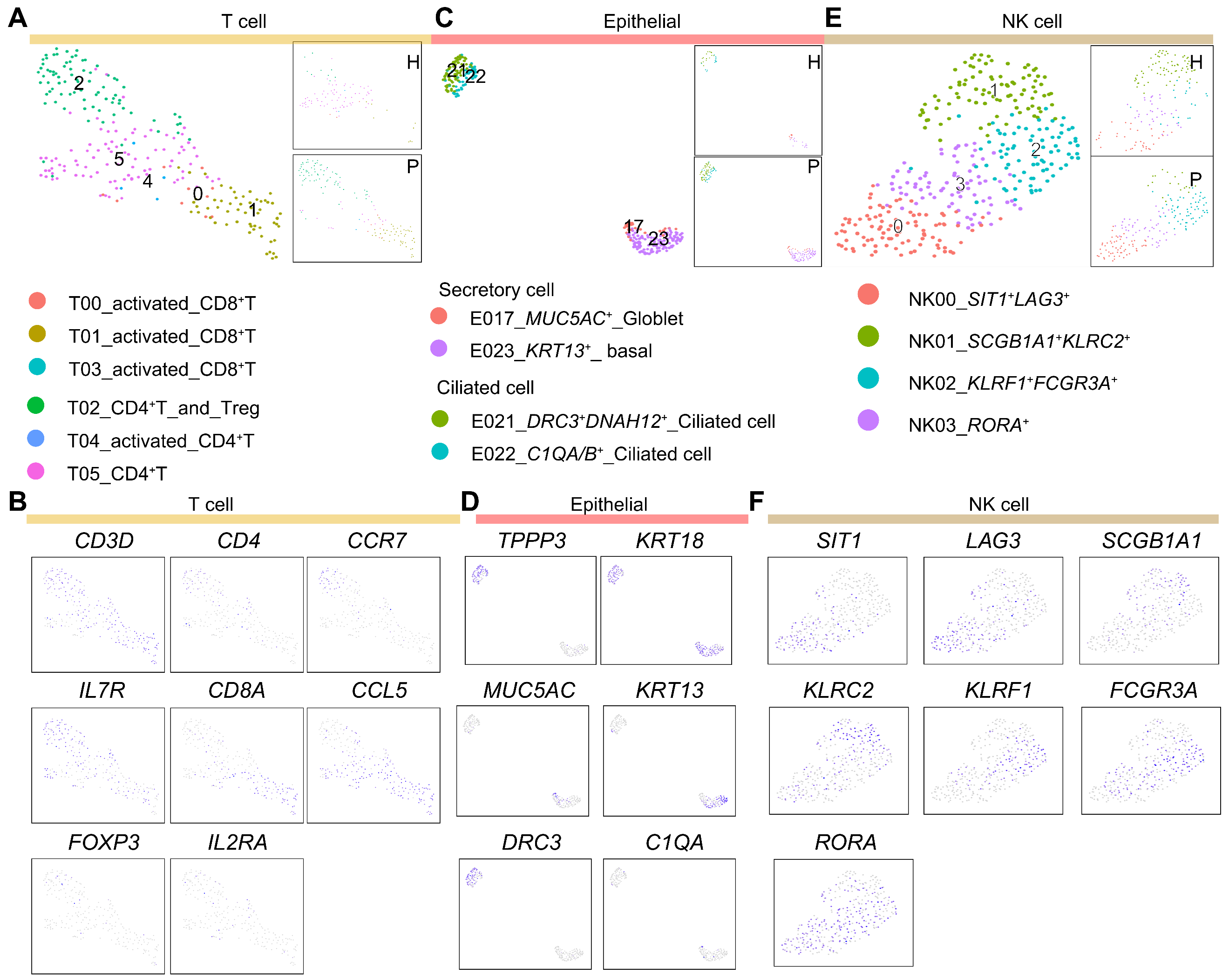


**Figure S8. Inflammatory cells and immune markers analysis in surviving mice.** (A) The proportion of macrophages in BALF of surviving mice 6 hours after PA intranasal inoculation (for each group, n=4). (B) Neutrophil counts in peripheral blood and BALF of surviving mice 6 hours after PA intranasal inoculation (for each group, n=4). (C) CXCR2 MFI in neutrophils in peripheral blood and BALF of surviving mice 6 hours after PA intranasal inoculation (for each group, n=4). (D) PD-L1 MFI in neutrophils peripheral blood and BALF of surviving mice 6 hours after PA intranasal inoculation (for each group, n=4). (E) Immunofluorescence staining for PD-L1 on neutrophils and PD-1 on CD4^+^ T cells in the spleen of CLP mice and Sham mice (scale bar 10 μm) (Red, Ly6G; green, PD-L1; yellow, CD4; cyan, PD-1). ns denotes not significant, * denotes p<0.05, ** denotes p<0.01, *** denotes p<0.001, by Student’s t test.


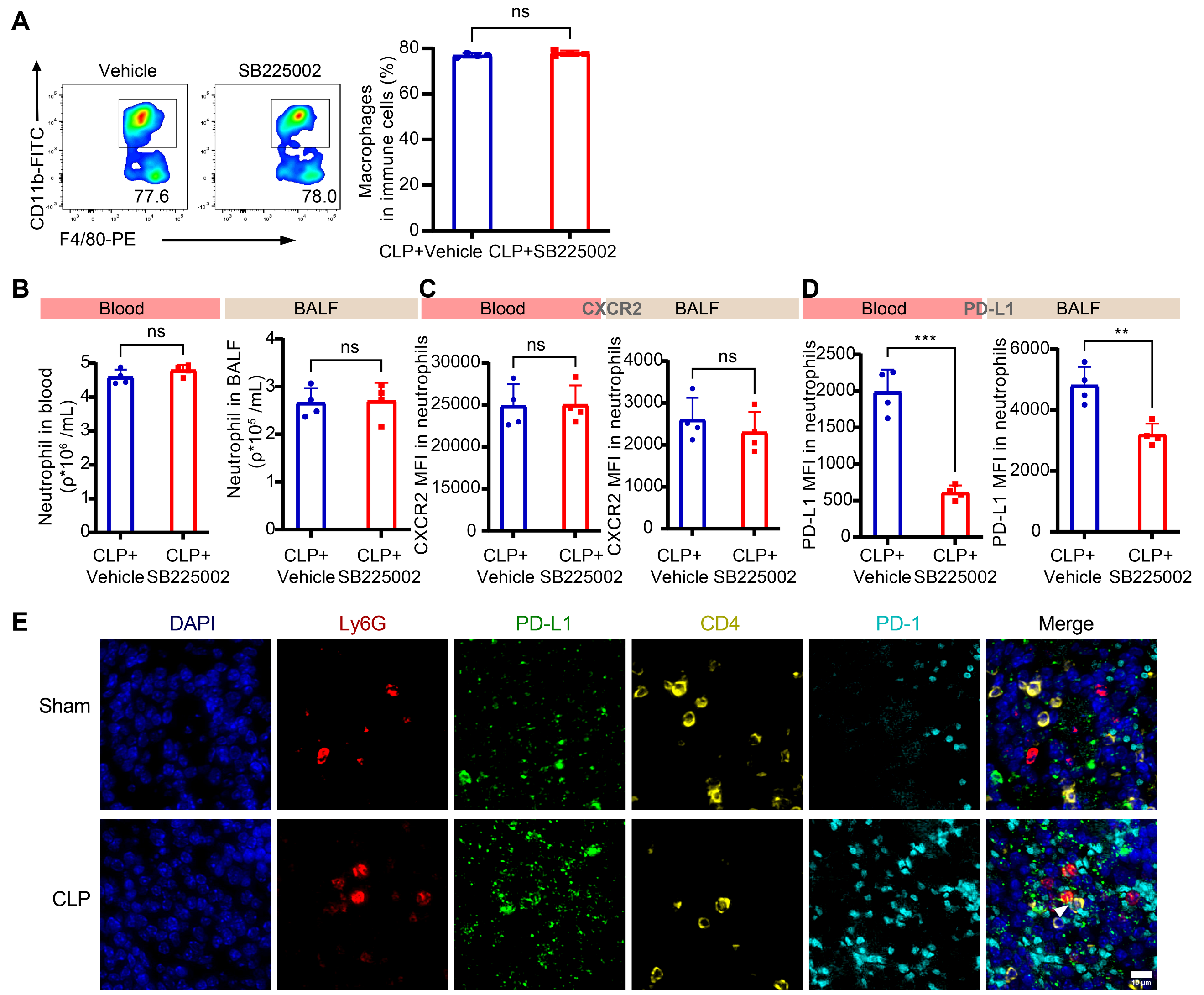


**Figure S9. Raw western blot images for this study.** (A-B) Raw images of PD-L1 and GAPDH expression corresponding to Figure 7B, D, E. (C-D) Raw images of PD-L1 and GAPDH expression corresponding to Figure 7H, J, K.


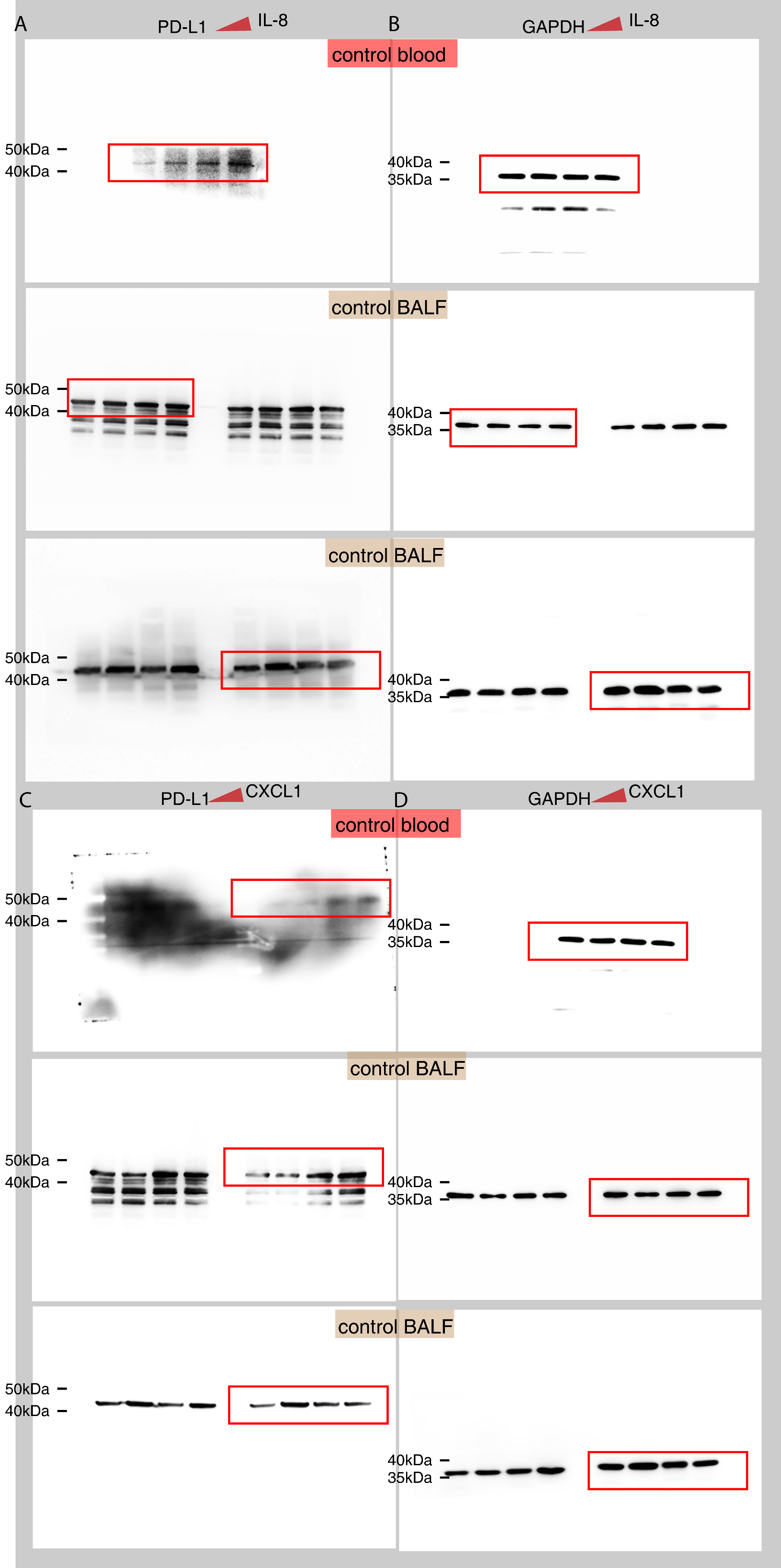


**Figure S10. Flow cytometry gating strategy for this study.** (A) Flow cytometry gating strategy for Figure 1F-H, 2J-K, 8B. (B) Flow cytometry gating strategy for Figure 5C-F, 7F-G, 7L-M, S4A-D. (C) Flow cytometry gating strategy for Figure 7P-Q, 8L-M. (D) Flow cytometry gating strategy for Figure 5G-J, 8C-D, S8C-D. (E) Flow cytometry gating strategy for Figure 2E-I. (F) Flow cytometry gating strategy for Figure S1A-C.


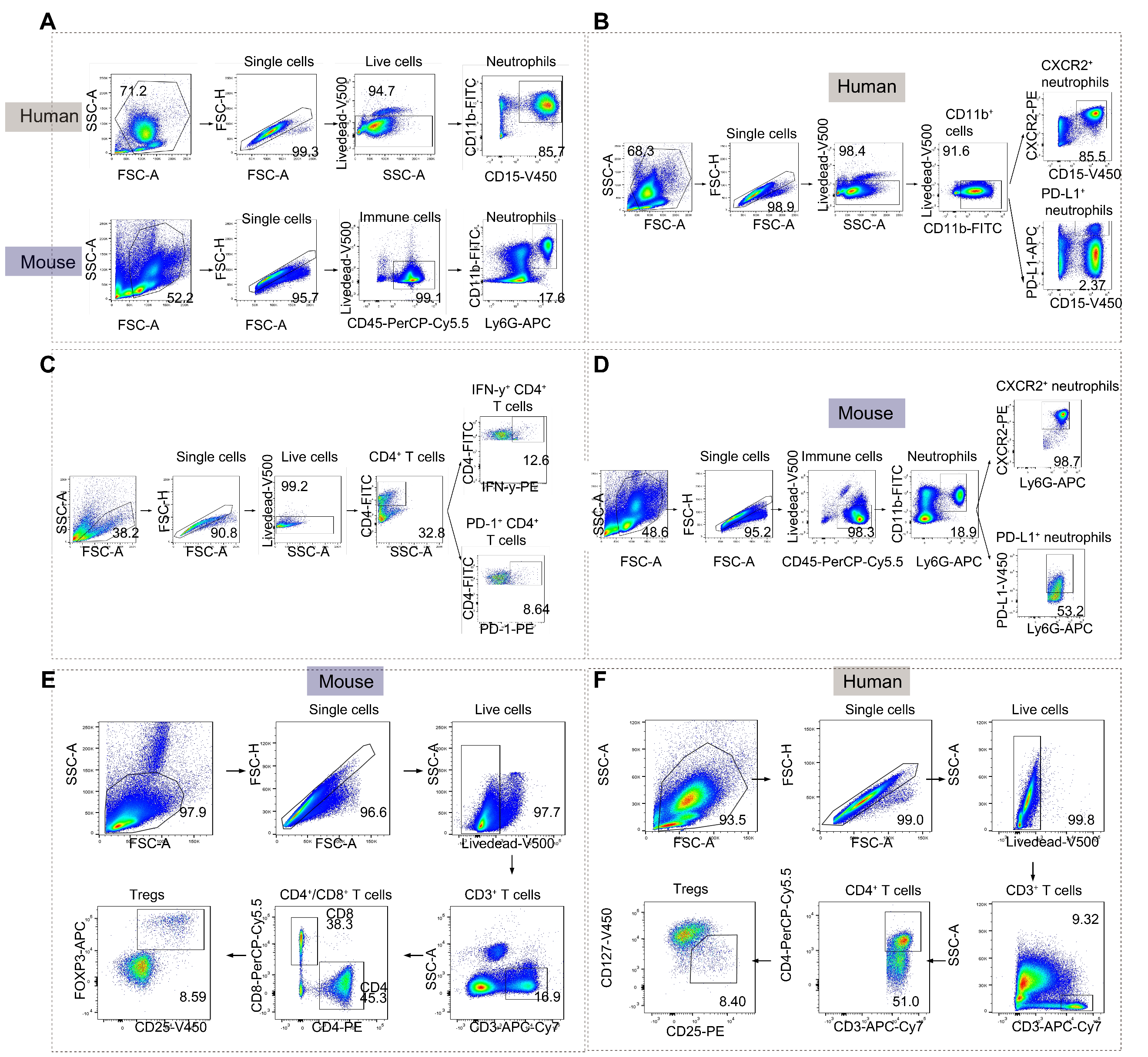


**Supplementary tables:**

**Table S1. Analysis characteristics of scRNA-seq samples involved in this study.**

| NO. | Sample | Status | Number of cells detected |
| --- | --- | --- | --- |
| P1 | BALF | Patient | 1552 |
| P2 | BALF | Patient | 1086 |
| P3 | BALF | Patient | 709 |
| P4 | BALF | Patient | 3922 |
| H1 | BALF | Healthy | 8529 |
| H2 | BALF | Healthy | 11112 |
| H3 | BALF | Healthy | 10361 |
| H4 | BALF | Healthy | 6583 |

**Table S2. Clinical characteristics of scRNA-seq samples involved in this study.**

|  | Sepsis patients (n = 4) | Healthy controls (n = 4) |
| --- | --- | --- |
| Age | 60 (12) | 29 (7) |
| Sex, male | 3 (75%) | 2 (50%) |
| SOFA score | 9.8 (2.4) | - |
| Mortality (14d) | 0 (0%) | - |
| Mortality (28d) | 3 (75%) | - |
| *Infection source* |  |  |
| Intra-abdominal sepsis | 3 (75%) | - |
| Meningitis | 1 (25%) | - |
| *Microbiology* |  |  |
| E. coli | 2 (50%) | - |
| S. aureus | 1 (25%) | - |
| Unknown | 1 (25%) | - |
| Intensive care (at point of sampling) | 4 (100%) | - |
| Fluid resuscitation | 4 (100%) | - |
| Nutrition support therapy | 4 (100%) | - |
| Antimicrobial therapy | 4 (100%) | - |
| Glucose management | 0 (0%) | - |
| Ulinastatin | 0 (0%) | - |
| Vasopressors | 4 (100%) | - |
| Blood glucose management | 4 (100%) | - |
| Mechanical ventilation | 4 (100%) | - |
| Renal replacement therapy | 0 (0%) | - |
| White cell count | 15.1 (2.1) | - |
| Proportion neutrophils | 87% | - |
| Proportion lymphocytes | 5% | - |
| Proportion monocytes | 4% | - |
| Proportion eosinophils | 1% | - |
| Days from hospital admission until sampling | 1.2 (0.6) | - |

Note: 1. Data are n (%) or mean (SD) unless otherwise specified. 2. SOFA=Sequential Organ Failure Assessment on day of sampling.

**Table S3. Clinical characteristics of experiment verification human samples involved in this study.**

|  | Sepsis patients (n = 15) | Healthy control (n = 15) |
| --- | --- | --- |
| Age | 67 (19) | 62 (12) |
| Sex, male | 9 (60%) | 6 (40%) |
| SOFA score | 7.6 (2.3) | - |
| Mortality (14d) | 0 (0%) | - |
| Mortality (28d) | 4 (27%) | - |
| Infection source |  |  |
| Community acquired pneumonia | 7 (46%) | - |
| Urosepsis | 3 (20%) | - |
| Intra-abdominal sepsis | 2 (13%) | - |
| Meningitis | 2 (13%) | - |
| Necrotising fasciitis | 1 (7%) | - |
| Intensive care (at point of sampling) | 15 (100%) |  |
| Fluid resuscitation | 15 (100%) | - |
| Nutrition support therapy | 15 (100%) | - |
| Antimicrobial therapy | 13 (87%) | - |
| Glucose management | 0 (0%) | - |
| Ulinastatin | 0 (0%) | - |
| Vasopressors | 15 (100%) | - |
| Blood glucose management | 15 (100%) | - |
| Mechanical ventilation | 14 (93%) | - |
| Renal replacement therapy | 1 (7%) | - |
| White cell count | 15.8 (9.6) | - |
| Proportion neutrophils | 86% |  |
| Proportion lymphocytes | 8% |  |
| Proportion monocytes | 5% |  |
| Proportion eosinophils | 1% |  |

Note: Data are n (%) or mean (SD) unless otherwise specified. SOFA=Sequential Organ Failure Assessment on day of sampling.

**Table S4. Characteristics of experiment verification mouse samples involved in this study.**

| NO. | Sample | Status | Gender | Age | Weight (g) | Days after surgery (d) | Intraperitoneal injection | Intranasal inoculation |
| --- | --- | --- | --- | --- | --- | --- | --- | --- |
| M1 | Blood | Sham | Male | 9 weeks | 22.9 | 1 | - | - |
| M2 | Blood | Sham | Male | 9 weeks | 22.4 | 1 | - | - |
| M3 | Blood | Sham | Male | 9 weeks | 20.6 | 1 | - | - |
| M4 | Blood | Sham | Male | 9 weeks | 21.3 | 1 | - | - |
| M5 | Blood | Sham | Male | 9 weeks | 23.6 | 1 | - | - |
| M6 | Blood | CLP | Male | 9 weeks | 24.6 | 1 | - | - |
| M7 | Blood | CLP | Male | 9 weeks | 22.9 | 1 | - | - |
| M8 | Blood | CLP | Male | 9 weeks | 24.3 | 1 | - | - |
| M9 | Blood | CLP | Male | 9 weeks | 22.1 | 1 | - | - |
| M10 | Blood | CLP | Male | 9 weeks | 23.5 | 1 | - | - |
| M11 | Blood | Sham | Male | 9 weeks | 23.6 | 3 | - | - |
| M12 | Blood | Sham | Male | 9 weeks | 23.1 | 3 | - | - |
| M13 | Blood | Sham | Male | 9 weeks | 22.5 | 3 | - | - |
| M14 | Blood | Sham | Male | 9 weeks | 20.9 | 3 | - | - |
| M15 | Blood | Sham | Male | 9 weeks | 21.9 | 3 | - | - |
| M16 | Blood | CLP | Male | 9 weeks | 23.9 | 3 | - | - |
| M17 | Blood | CLP | Male | 9 weeks | 21.1 | 3 | - | - |
| M18 | Blood | CLP | Male | 9 weeks | 22.8 | 3 | - | - |
| M19 | Blood | CLP | Male | 9 weeks | 20.4 | 3 | - | - |
| M20 | Blood | CLP | Male | 9 weeks | 21.8 | 3 | - | - |
| M21 | Blood | Sham | Male | 9 weeks | 23.9 | 7 | - | - |
| M22 | Blood | Sham | Male | 9 weeks | 23.9 | 7 | - | - |
| M23 | Blood | Sham | Male | 9 weeks | 23.2 | 7 | - | - |
| M24 | Blood | Sham | Male | 9 weeks | 23.1 | 7 | - | - |
| M25 | Blood | Sham | Male | 9 weeks | 21.8 | 7 | - | - |
| M26 | Blood | CLP | Male | 9 weeks | 24.3 | 7 | - | - |
| M27 | Blood | CLP | Male | 9 weeks | 20.8 | 7 | - | - |
| M28 | Blood | CLP | Male | 9 weeks | 23.1 | 7 | - | - |
| M29 | Blood | CLP | Male | 9 weeks | 23.2 | 7 | - | - |
| M30 | Blood | CLP | Male | 9 weeks | 20.3 | 7 | - | - |
| M1 | BALF | Sham | Male | 9 weeks | 22.9 | 1 | - | - |
| M2 | BALF | Sham | Male | 9 weeks | 22.4 | 1 | - | - |
| M3 | BALF | Sham | Male | 9 weeks | 20.6 | 1 | - | - |
| M4 | BALF | Sham | Male | 9 weeks | 21.3 | 1 | - | - |
| M5 | BALF | Sham | Male | 9 weeks | 23.6 | 1 | - | - |
| M6 | BALF | CLP | Male | 9 weeks | 24.6 | 1 | - | - |
| M7 | BALF | CLP | Male | 9 weeks | 22.9 | 1 | - | - |
| M8 | BALF | CLP | Male | 9 weeks | 24.3 | 1 | - | - |
| M9 | BALF | CLP | Male | 9 weeks | 22.1 | 1 | - | - |
| M10 | BALF | CLP | Male | 9 weeks | 23.5 | 1 | - | - |
| M11 | BALF | Sham | Male | 9 weeks | 23.6 | 3 | - | - |
| M12 | BALF | Sham | Male | 9 weeks | 23.1 | 3 | - | - |
| M13 | BALF | Sham | Male | 9 weeks | 22.5 | 3 | - | - |
| M14 | BALF | Sham | Male | 9 weeks | 20.9 | 3 | - | - |
| M15 | BALF | Sham | Male | 9 weeks | 21.9 | 3 | - | - |
| M16 | BALF | CLP | Male | 9 weeks | 23.9 | 3 | - | - |
| M17 | BALF | CLP | Male | 9 weeks | 21.1 | 3 | - | - |
| M18 | BALF | CLP | Male | 9 weeks | 22.8 | 3 | - | - |
| M19 | BALF | CLP | Male | 9 weeks | 20.4 | 3 | - | - |
| M20 | BALF | CLP | Male | 9 weeks | 21.8 | 3 | - | - |
| M21 | BALF | Sham | Male | 9 weeks | 23.9 | 7 | - | - |
| M22 | BALF | Sham | Male | 9 weeks | 23.9 | 7 | - | - |
| M23 | BALF | Sham | Male | 9 weeks | 23.2 | 7 | - | - |
| M24 | BALF | Sham | Male | 9 weeks | 23.1 | 7 | - | - |
| M25 | BALF | Sham | Male | 9 weeks | 21.8 | 7 | - | - |
| M26 | BALF | CLP | Male | 9 weeks | 24.3 | 7 | - | - |
| M27 | BALF | CLP | Male | 9 weeks | 20.8 | 7 | - | - |
| M28 | BALF | CLP | Male | 9 weeks | 23.1 | 7 | - | - |
| M29 | BALF | CLP | Male | 9 weeks | 23.2 | 7 | - | - |
| M30 | BALF | CLP | Male | 9 weeks | 20.3 | 7 | - | - |
| M31 | Blood | Sham | Male | 10 weeks | 22.5 | 1 | - | - |
| M32 | Blood | Sham | Male | 10 weeks | 23.9 | 1 | - | - |
| M33 | Blood | Sham | Male | 10 weeks | 24.1 | 1 | - | - |
| M34 | Blood | Sham | Male | 10 weeks | 24.5 | 1 | - | - |
| M35 | Blood | Sham | Male | 10 weeks | 25.1 | 1 | - | - |
| M36 | Blood | CLP | Male | 10 weeks | 20.8 | 1 | - | - |
| M37 | Blood | CLP | Male | 10 weeks | 22.9 | 1 | - | - |
| M38 | Blood | CLP | Male | 10 weeks | 22.0 | 1 | - | - |
| M39 | Blood | CLP | Male | 10 weeks | 21.6 | 1 | - | - |
| M40 | Blood | CLP | Male | 10 weeks | 20.6 | 1 | - | - |
| M41 | Blood | Sham | Male | 10 weeks | 24.7 | 3 | - | - |
| M42 | Blood | Sham | Male | 10 weeks | 24.5 | 3 | - | - |
| M43 | Blood | Sham | Male | 10 weeks | 24.3 | 3 | - | - |
| M44 | Blood | Sham | Male | 10 weeks | 22.8 | 3 | - | - |
| M45 | Blood | Sham | Male | 10 weeks | 23.0 | 3 | - | - |
| M46 | Blood | CLP | Male | 10 weeks | 21.6 | 3 | - | - |
| M47 | Blood | CLP | Male | 10 weeks | 23.4 | 3 | - | - |
| M48 | Blood | CLP | Male | 10 weeks | 21.3 | 3 | - | - |
| M49 | Blood | CLP | Male | 10 weeks | 23.2 | 3 | - | - |
| M50 | Blood | CLP | Male | 10 weeks | 21.4 | 3 | - | - |
| M51 | Blood | Sham | Male | 10 weeks | 24.0 | 7 | - | - |
| M52 | Blood | Sham | Male | 10 weeks | 24.4 | 7 | - | - |
| M53 | Blood | Sham | Male | 10 weeks | 22.5 | 7 | - | - |
| M54 | Blood | Sham | Male | 10 weeks | 23.6 | 7 | - | - |
| M55 | Blood | Sham | Male | 10 weeks | 24.8 | 7 | - | - |
| M56 | Blood | CLP | Male | 10 weeks | 23.7 | 7 | - | - |
| M57 | Blood | CLP | Male | 10 weeks | 21.0 | 7 | - | - |
| M58 | Blood | CLP | Male | 10 weeks | 22.2 | 7 | - | - |
| M59 | Blood | CLP | Male | 10 weeks | 20.1 | 7 | - | - |
| M60 | Blood | CLP | Male | 10 weeks | 20.5 | 7 | - | - |
| M31 | BALF | Sham | Male | 10 weeks | 22.5 | 1 | - | - |
| M32 | BALF | Sham | Male | 10 weeks | 23.9 | 1 | - | - |
| M33 | BALF | Sham | Male | 10 weeks | 24.1 | 1 | - | - |
| M34 | BALF | Sham | Male | 10 weeks | 24.5 | 1 | - | - |
| M35 | BALF | Sham | Male | 10 weeks | 25.1 | 1 | - | - |
| M36 | BALF | CLP | Male | 10 weeks | 20.8 | 1 | - | - |
| M37 | BALF | CLP | Male | 10 weeks | 22.9 | 1 | - | - |
| M38 | BALF | CLP | Male | 10 weeks | 22.0 | 1 | - | - |
| M39 | BALF | CLP | Male | 10 weeks | 21.6 | 1 | - | - |
| M40 | BALF | CLP | Male | 10 weeks | 20.6 | 1 | - | - |
| M41 | BALF | Sham | Male | 10 weeks | 24.7 | 3 | - | - |
| M42 | BALF | Sham | Male | 10 weeks | 24.5 | 3 | - | - |
| M43 | BALF | Sham | Male | 10 weeks | 24.3 | 3 | - | - |
| M44 | BALF | Sham | Male | 10 weeks | 22.8 | 3 | - | - |
| M45 | BALF | Sham | Male | 10 weeks | 23.0 | 3 | - | - |
| M46 | BALF | CLP | Male | 10 weeks | 21.6 | 3 | - | - |
| M47 | BALF | CLP | Male | 10 weeks | 23.4 | 3 | - | - |
| M48 | BALF | CLP | Male | 10 weeks | 21.3 | 3 | - | - |
| M49 | BALF | CLP | Male | 10 weeks | 23.2 | 3 | - | - |
| M50 | BALF | CLP | Male | 10 weeks | 21.4 | 3 | - | - |
| M51 | BALF | Sham | Male | 10 weeks | 24.0 | 7 | - | - |
| M52 | BALF | Sham | Male | 10 weeks | 24.4 | 7 | - | - |
| M53 | BALF | Sham | Male | 10 weeks | 22.5 | 7 | - | - |
| M54 | BALF | Sham | Male | 10 weeks | 23.6 | 7 | - | - |
| M55 | BALF | Sham | Male | 10 weeks | 24.8 | 7 | - | - |
| M56 | BALF | CLP | Male | 10 weeks | 23.7 | 7 | - | - |
| M57 | BALF | CLP | Male | 10 weeks | 21.0 | 7 | - | - |
| M58 | BALF | CLP | Male | 10 weeks | 22.2 | 7 | - | - |
| M59 | BALF | CLP | Male | 10 weeks | 20.1 | 7 | - | - |
| M60 | BALF | CLP | Male | 10 weeks | 20.5 | 7 | - | - |
| M61 | Blood | Sham | Male | 9 weeks | 23.5 | 7 | Vehicle | PA |
| M62 | Blood | Sham | Male | 9 weeks | 23.5 | 7 | Vehicle | PA |
| M63 | Blood | Sham | Male | 9 weeks | 25 | 7 | Vehicle | PA |
| M64 | Blood | CLP | Male | 9 weeks | 24.8 | 7 | Vehicle | PA |
| M65 | Blood | CLP | Male | 9 weeks | 20.9 | 7 | Vehicle | PA |
| M66 | Blood | CLP | Male | 9 weeks | 22.1 | 7 | Vehicle | PA |
| M67 | Blood | CLP | Male | 9 weeks | 22.7 | 7 | Vehicle | PA |
| M68 | Blood | Sham | Male | 9 weeks | 23.2 | 7 | SB225002 | PA |
| M69 | Blood | Sham | Male | 9 weeks | 22.5 | 7 | SB225002 | PA |
| M70 | Blood | Sham | Male | 9 weeks | 24.4 | 7 | SB225002 | PA |
| M71 | Blood | CLP | Male | 9 weeks | 25.3 | 7 | SB225002 | PA |
| M72 | Blood | CLP | Male | 9 weeks | 21.1 | 7 | SB225002 | PA |
| M73 | Blood | CLP | Male | 9 weeks | 23.2 | 7 | SB225002 | PA |
| M74 | Blood | CLP | Male | 9 weeks | 23.2 | 7 | SB225002 | PA |
| M61 | BALF | Sham | Male | 9 weeks | 23.5 | 7 | Vehicle | PA |
| M62 | BALF | Sham | Male | 9 weeks | 23.5 | 7 | Vehicle | PA |
| M63 | BALF | Sham | Male | 9 weeks | 25 | 7 | Vehicle | PA |
| M64 | BALF | CLP | Male | 9 weeks | 24.8 | 7 | Vehicle | PA |
| M65 | BALF | CLP | Male | 9 weeks | 20.9 | 7 | Vehicle | PA |
| M66 | BALF | CLP | Male | 9 weeks | 22.1 | 7 | Vehicle | PA |
| M67 | BALF | CLP | Male | 9 weeks | 22.7 | 7 | Vehicle | PA |
| M68 | BALF | Sham | Male | 9 weeks | 23.2 | 7 | SB225002 | PA |
| M69 | BALF | Sham | Male | 9 weeks | 22.5 | 7 | SB225002 | PA |
| M70 | BALF | Sham | Male | 9 weeks | 24.4 | 7 | SB225002 | PA |
| M71 | BALF | CLP | Male | 9 weeks | 25.3 | 7 | SB225002 | PA |
| M72 | BALF | CLP | Male | 9 weeks | 21.1 | 7 | SB225002 | PA |
| M73 | BALF | CLP | Male | 9 weeks | 23.2 | 7 | SB225002 | PA |
| M74 | BALF | CLP | Male | 9 weeks | 23.2 | 7 | SB225002 | PA |
| M75 | Lung | Sham | Male | 9 weeks | 22.6 | 7 | Vehicle | PA |
| M76 | Lung | Sham | Male | 9 weeks | 25.0 | 7 | Vehicle | PA |
| M77 | Lung | Sham | Male | 9 weeks | 24.5 | 7 | Vehicle | PA |
| M78 | Lung | CLP | Male | 9 weeks | 22.5 | 7 | Vehicle | PA |
| M79 | Lung | CLP | Male | 9 weeks | 21.5 | 7 | Vehicle | PA |
| M80 | Lung | CLP | Male | 9 weeks | 20.7 | 7 | Vehicle | PA |
| M81 | Lung | Sham | Male | 9 weeks | 23.3 | 7 | SB225002 | PA |
| M82 | Lung | Sham | Male | 9 weeks | 23.8 | 7 | SB225002 | PA |
| M83 | Lung | Sham | Male | 9 weeks | 24.2 | 7 | SB225002 | PA |
| M84 | Lung | CLP | Male | 9 weeks | 22.7 | 7 | SB225002 | PA |
| M85 | Lung | CLP | Male | 9 weeks | 21.5 | 7 | SB225002 | PA |
| M86 | Lung | CLP | Male | 9 weeks | 21.6 | 7 | SB225002 | PA |
| M75 | Liver | Sham | Male | 9 weeks | 22.6 | 7 | Vehicle | PA |
| M76 | Liver | Sham | Male | 9 weeks | 25.0 | 7 | Vehicle | PA |
| M77 | Liver | Sham | Male | 9 weeks | 24.5 | 7 | Vehicle | PA |
| M78 | Liver | CLP | Male | 9 weeks | 22.5 | 7 | Vehicle | PA |
| M79 | Liver | CLP | Male | 9 weeks | 21.5 | 7 | Vehicle | PA |
| M80 | Liver | CLP | Male | 9 weeks | 20.7 | 7 | Vehicle | PA |
| M81 | Liver | Sham | Male | 9 weeks | 23.3 | 7 | SB225002 | PA |
| M82 | Liver | Sham | Male | 9 weeks | 23.8 | 7 | SB225002 | PA |
| M83 | Liver | Sham | Male | 9 weeks | 24.2 | 7 | SB225002 | PA |
| M84 | Liver | CLP | Male | 9 weeks | 22.7 | 7 | SB225002 | PA |
| M85 | Liver | CLP | Male | 9 weeks | 21.5 | 7 | SB225002 | PA |
| M86 | Liver | CLP | Male | 9 weeks | 21.6 | 7 | SB225002 | PA |
| M75 | Kidney | Sham | Male | 9 weeks | 22.6 | 7 | Vehicle | PA |
| M76 | Kidney | Sham | Male | 9 weeks | 25.0 | 7 | Vehicle | PA |
| M77 | Kidney | Sham | Male | 9 weeks | 24.5 | 7 | Vehicle | PA |
| M78 | Kidney | CLP | Male | 9 weeks | 22.5 | 7 | Vehicle | PA |
| M79 | Kidney | CLP | Male | 9 weeks | 21.5 | 7 | Vehicle | PA |
| M80 | Kidney | CLP | Male | 9 weeks | 20.7 | 7 | Vehicle | PA |
| M81 | Kidney | Sham | Male | 9 weeks | 23.3 | 7 | SB225002 | PA |
| M82 | Kidney | Sham | Male | 9 weeks | 23.8 | 7 | SB225002 | PA |
| M83 | Kidney | Sham | Male | 9 weeks | 24.2 | 7 | SB225002 | PA |
| M84 | Kidney | CLP | Male | 9 weeks | 22.7 | 7 | SB225002 | PA |
| M85 | Kidney | CLP | Male | 9 weeks | 21.5 | 7 | SB225002 | PA |
| M86 | Kidney | CLP | Male | 9 weeks | 21.6 | 7 | SB225002 | PA |
| M87 | - | Sham | Male | 9 weeks | 24.2 | 10 | Vehicle | PA |
| M88 | - | Sham | Male | 9 weeks | 22.5 | 10 | Vehicle | PA |
| M89 | - | Sham | Male | 9 weeks | 24.4 | 10 | Vehicle | PA |
| M90 | - | Sham | Male | 9 weeks | 24.6 | 10 | Vehicle | PA |
| M91 | - | Sham | Male | 9 weeks | 24.7 | 10 | Vehicle | PA |
| M92 | - | Sham | Male | 9 weeks | 23.2 | 10 | Vehicle | PA |
| M93 | - | CLP | Male | 9 weeks | 22.1 | 10 | Vehicle | PA |
| M94 | - | CLP | Male | 9 weeks | 24.6 | 10 | Vehicle | PA |
| M95 | - | CLP | Male | 9 weeks | 22.0 | 10 | Vehicle | PA |
| M96 | - | CLP | Male | 9 weeks | 24.0 | 10 | Vehicle | PA |
| M97 | - | CLP | Male | 9 weeks | 20.3 | 10 | Vehicle | PA |
| M98 | - | CLP | Male | 9 weeks | 20.0 | 10 | Vehicle | PA |
| M99 | - | CLP | Male | 9 weeks | 21.6 | 10 | Vehicle | PA |
| M100 | - | CLP | Male | 9 weeks | 24.4 | 10 | Vehicle | PA |
| M101 | - | CLP | Male | 9 weeks | 21.9 | 10 | Vehicle | PA |
| M102 | - | CLP | Male | 9 weeks | 23.2 | 10 | Vehicle | PA |
| M103 | - | Sham | Male | 9 weeks | 23.1 | 10 | SB225002 | PA |
| M104 | - | Sham | Male | 9 weeks | 24.4 | 10 | SB225002 | PA |
| M105 | - | Sham | Male | 9 weeks | 24.8 | 10 | SB225002 | PA |
| M106 | - | Sham | Male | 9 weeks | 24.8 | 10 | SB225002 | PA |
| M107 | - | Sham | Male | 9 weeks | 25.0 | 10 | SB225002 | PA |
| M108 | - | Sham | Male | 9 weeks | 24.9 | 10 | SB225002 | PA |
| M109 | - | CLP | Male | 9 weeks | 24.6 | 10 | SB225002 | PA |
| M110 | - | CLP | Male | 9 weeks | 21.2 | 10 | SB225002 | PA |
| M111 | - | CLP | Male | 9 weeks | 24.0 | 10 | SB225002 | PA |
| M112 | - | CLP | Male | 9 weeks | 22.7 | 10 | SB225002 | PA |
| M113 | - | CLP | Male | 9 weeks | 24.8 | 10 | SB225002 | PA |
| M114 | - | CLP | Male | 9 weeks | 21.4 | 10 | SB225002 | PA |
| M115 | - | CLP | Male | 9 weeks | 22.6 | 10 | SB225002 | PA |
| M116 | - | CLP | Male | 9 weeks | 23.2 | 10 | SB225002 | PA |
| M117 | - | CLP | Male | 9 weeks | 21.9 | 10 | SB225002 | PA |
| M118 | - | CLP | Male | 9 weeks | 23.8 | 10 | SB225002 | PA |

**Supplementary information**

**Heterogeneity of macrophages**: In late-stage immunosuppressive sepsis BALF, the proportion of macrophage cells was significantly decreased (p<0.01), while macrophages showed great heterogeneity in cell composition between sepsis patients and controls (**Figure 1B, 1D**). Re-clustering of 33,614 macrophages found that, according to the differences in gene expression, the macrophages in the BALF of late-stage immunosuppressive sepsis patients and healthy controls could be further divided into M00-M07 types (**Figure S6A-C**). It is worth mentioning that in patients, the proportion of *CXCL8^+^CCL4^+^CCL3^+^* M02 cells was significantly increased (**Figure S6D**; p<0.05), while *CXCL8*^+^*IFITM1*^+^ M04 also showed an increasing trend (p<0.1), indicating that M02 and M04 mainly exist in late-stage immunosuppressive sepsis patients and may be more related to sepsis. Both M02/M04 subpopulations highly express *CXCL8*. In addition, there was no significant change in the proportion of M00, M03, M05, M06, and M07 in patients (**Figure S6D**, p>0.1), while the proportion of M01 showed a downward trend (p<0.1), suggesting that these subpopulations may be less related to late-stage immunosuppressive sepsis. Differential sepsis-specific gene expression analysis of macrophages found that *CXCL8* was significantly upregulated in patients in M02 and M04 (**Figure S6F**). Macrophages are usually divided into M1 type (*HLA-DR*^+^, *CCR7*^+^) and M2 type (*CD163^+^CD209^+^MRC1^+^CCL2*^+^) according to their proinflammatory/anti-inflammatory properties. However, M02/M04 did not specifically express M1/M2-type markers (**Figure S6E**) compared to other macrophage subpopulations. Since M02/M04 was significantly increased in the BALF of patients with late-stage immunosuppressive sepsis, unlike other subpopulations, *CXCL8*^+^ M02 and M04 could be a specific macrophage subpopulation in the late-stage immunosuppressive sepsis BALF environment.

**Heterogeneity of epithelial cells:** BALF epithelial cells in late-stage immunosuppressive sepsis patients were further divided into 4 subpopulations by signature genes: E017 (MUC5AC^+^), E023 (KRT13^+^), E021 (DRC3^+^), and E022 (C1QA^+^) (**Figure S7C-D**). Based on the expression of signature genes, E017 and E023 were globlet and basel secretory cells, and E021 and E022 were ciliated cells. The proportions of E017, E021 and E022 showed a decreasing trend in patients, while E023 displayed an increasing trend in patients (**Figure S7C**).

**Heterogeneity of T cells:** BALF T cells in late-stage immunosuppressive sepsis patients were further divided into 6 subpopulations by signature genes: T00, T01, and T03 were CD8^+^ T cells, and T02, T04, and T05 were CD4^+^ T cells based on the expression of signature genes (**Figure S7A-B**). The proportion of all T-cell subpopulations showed a decreasing trend in patients, except for T02, which contained CD4^+^ and Treg cells, which did not show a decreasing trend in patients (**Figure S7A**).

**Heterogeneity of NK cells:** BALF NK cells in late-stage immunosuppressive sepsis patients were further divided into 4 subpopulations by signature genes, NK00, NK01, NK02, and NK03, based on the expression of signature genes (**Figure S7E-F**).
